# Cardiac surgery on patients with COVID‐19: a systematic review and meta‐analysis

**DOI:** 10.1111/ans.17667

**Published:** 2022-04-03

**Authors:** Aashray K. Gupta, Alasdair Leslie, Joseph N. Hewitt, Joshua G. Kovoor, Christopher D. Ovenden, Suzanne Edwards, Justin C. Y. Chan, Michael G. Worthington

**Affiliations:** ^1^ Department of Surgery University of Sydney Sydney New South Wales Australia; ^2^ Department of Cardiothoracic Surgery Royal Adelaide Hospital Adelaide Australia; ^3^ University of Adelaide, Discipline of Surgery Royal Adelaide Hospital Adelaide South Australia Australia; ^4^ Adelaide Health Technology Assessment, School of Public Health University of Adelaide Adelaide South Australia Australia

**Keywords:** aortic dissection, bypass grafting, cardiac surgery, coronary artery, COVID‐19, emergency surgery

## Abstract

**Introduction:**

The COVID‐19 pandemic has had a significant impact on global surgery. In particular, deleterious effects of SARS‐CoV‐2 infection on the heart and cardiovascular system have been described. To inform surgical patients, we performed a systematic review and meta‐analysis aiming to characterize outcomes of COVID‐19 positive patients undergoing cardiac surgery.

**Methods:**

The study protocol was registered with PROSPERO (CRD42021228533) and conformed with PRISMA 2020 and MOOSE guidelines. PubMed, Ovid MEDLINE and Web of Science were searched between 1 January 2019 to 24 February 2022 for studies reporting outcomes on COVID‐19 positive patients undergoing cardiac surgery. Study screening, data extraction and risk of bias assessment were conducted in duplicate. Meta‐analysis was conducted using a random‐effects model where at least two studies had sufficient data for that variable.

**Results:**

Searches identified 4223 articles of which 18 studies were included with a total 44 patients undergoing cardiac surgery. Within these studies, 12 (66.7%) reported populations undergoing coronary artery bypass graft (CABG) surgery, three (16.7%) aortic valve replacements (AVR) and three (16.7%) aortic dissection repairs. Overall mean postoperative length of ICU stay was 3.39 (95% confidence interval (CI): 0.38, 6.39) and mean postoperative length of hospital stay was 17.88 (95% CI: 14.57, 21.19).

**Conclusion:**

This systematic review and meta‐analysis investigated studies of limited quality which characterized cardiac surgery in COVID‐19 positive patients and demonstrates that these patients have poor outcomes. Further issues to be explored are effects of COVID‐19 on decision‐making in cardiac surgery, and effects of COVID‐19 on the cardiovascular system at a cellular level.

## Graphical abstract

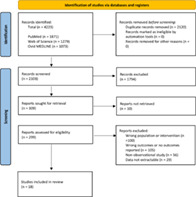

## Introduction

The unprecedented coronavirus (COVID‐19) pandemic, caused by severe acute respiratory syndrome coronavirus 2 (SARS‐CoV‐2), has had a dramatic effect on the global population.^1^ The advent of vaccines in late 2020 resulted in many government programs encouraging and at times mandating vaccinations for sections of the population. Increasing vaccination among elderly, immunocompromised and vulnerable people resulted in lower infections, hospitalizations and deaths from the virus. Governments which had implemented physical distancing policies to reduce transmission, proceeded to remove these restrictions in part or full.^2^ Recently, new variants of concern have provided a renewed threat due to their higher transmissibility and ability to evade vaccine defence.^3^, ^4^, ^5^, ^6^

Cardiac surgery comes with attendant risks. The decision to operate is based on the perceived risks and benefits discussed between a patient and surgeon. Patient factors are taken into account, along with operative considerations, clinical urgency and expected postoperative recovery.^7^, ^8^ Active infection with COVID‐19 represents a serious factor with the potential to cause morbidity and mortality. Various data regarding deleterious effects of CARS‐CoV‐2 in the heart and cardiovascular system have been reported.^9^, ^10^ To inform the decision‐making process in cardiac surgery worldwide, we performed a systematic review aiming to characterize the outcomes of COVID‐19 positive patients undergoing cardiac surgery.

## Methods

We performed a systematic review according to a protocol registered prior to commencement with the International Prospective Register of Systematic Reviews (PROSPERO, CRD42021228533). Our results were reported in accordance with the Preferred Reporting Items for Systematic Reviews and Meta‐Analyses (PRISMA) 2020 and the Meta‐Analysis of Observational Studies in Epidemiology (MOOSE) guidelines^11^, ^12^ (Data S1–S2).

### Search strategy and selection criteria

The population for included studies was patients who had COVID‐19 infection, defined as a positive laboratory diagnosis. The intervention was cardiac surgery. Where reported, the comparator was outcomes from cardiac surgical procedures in patients who did not have COVID‐19 infection. Reports of patients who were previously diagnosed with COVID‐19 but were deemed by the treating team to have cleared the infection were excluded. The primary outcome was mortality (both in‐hospital or 30‐day), while secondary outcomes included postoperative length of ICU and hospital stay, and postoperative complications including major adverse cardiovascular and cerebrovascular events.

PubMed, Ovid MEDLINE and Web of Science Core Collection databases were searched between 1 January 2019 and 24 February 2022. There were no other filters or restrictions applied. Search terms included (COVID OR ‘COVID‐19’ OR coronavirus OR ‘2019‐nCoV’ OR ‘SARS‐CoV‐2’) AND (‘cardiac surg*’ OR ‘cardiothoracic surg*’ OR ‘thoracic surg*’ OR ‘heart surg*’) AND (outcome* OR mortal*). These searches were supplemented by review of the grey literature, searching the bibliographies of included studies and targeted searches of Google Scholar and Scopus.

### Data extraction

After removal of duplicate items, studies were reviewed for inclusion by two independent investigators (JNH and AL). This was performed with a free‐to‐use web application (Rayyan, Qatar Computing Research Institute, Ar‐Rayyan, Qatar^13^). Studies were screened firstly by title and abstracts and subsequently full‐text reviews. Discrepancies were resolved by a third independent investigator (AKG). Extraction of Data was performed using a pre‐designed extraction form by two independent investigators (AKG and AL) and discrepancies resolved by consensus. Extracted data included study design, setting, population demographics, surgical intervention, postoperative length of ICU and hospital stay and outcomes. The extracted data were synthesized into narrative and tabular formats.

### Data analysis

Data analyses were performed using Stata Statistical Software: Release 15.1 College Station, TX: StataCorp LP. The I^2^ statistic was used to evaluate heterogeneity (with I^2^ >50% indicating significant heterogeneity) as was Cochran's Q P value (with *P*‐value <0.05 indicating significant heterogeneity). A random‐effects model was used throughout. A *P*‐value of <0.05 denoted statistical significance. A variable was included in the meta‐analysis if at least two journal articles involved had sufficient values for that variable. However, as many studies were case studies with one subject only, there were not two studies to compare when dividing the analysis into type of surgery subgroups: CABG, aortic dissection repair and aortic valve replacement. Due to only four valid studies for each comparison, Funnel plots and Eggers Tests would not be informative, and although attempted, meta‐regression was found to require more than four studies. The Downs and Black risk of bias checklist^14^ was used by two independent investigators (AKG and AL) to assess risk of bias and methodological quality of the included studies.

## Results

**Fig. 1:**
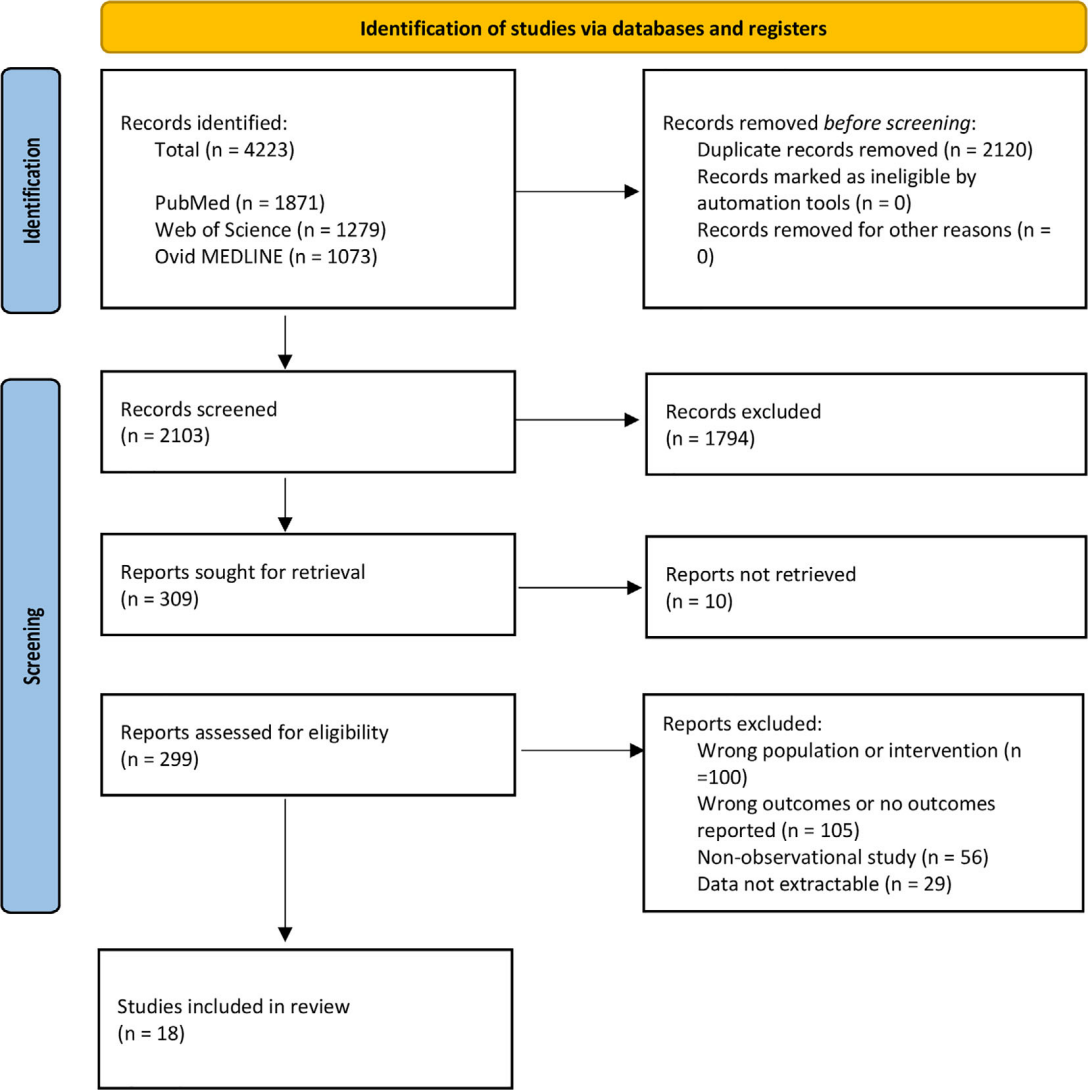
PRISMA diagram detailing the inclusion and exclusion of articles.

Our search strategy identified 4223 records which were screened for duplicates, with a total of 2103 articles progressing to title and abstract screening. Of these, 309 were relevant and proceeded to full‐text review (Data S3). Ten full texts were unable to be obtained, and so 299 articles were assessed for eligibility based on the inclusion criteria. From these, 100 had the wrong population group or intervention, 105 reported the wrong outcomes, 56 were non‐observational studies and 29 in which the data were not extractable. Accordingly, a total of 18^15^, ^16^, ^17^, ^18^, ^19^, ^20^, ^21^, ^22^, ^23^, ^24^, ^25^, ^26^, ^27^, ^28^, ^29^, ^30^, ^31^, ^32^ articles fit the inclusion criteria and were included in our systematic review and meta‐analysis. This is outlined in Fig. 1.

### Study characteristics

The characteristics of the 18 included studies are outlined in Table 1. Regarding the country of origin of the included studies, three were from the United States (USA, 27.8%), three from the United Kingdom (16.7%) and two from Italy (11.1%). The total sample size across the 18 studies was 44 patients undergoing cardiac surgery. Within the included studies, 12 (66.7%) reported populations undergoing coronary artery bypass graft (CABG) surgery, three (16.7%) aortic valve replacements (AVR) and three (16.7%) aortic dissection repairs. Regarding sex in the total population, the majority of patients were male (61.4%). When it was reported, mean age was 56.7 years. Four studies reported either society of thoracic surgeons (STS) score^33^ or European system for cardiac operative risk evaluation^34^ (EuroSCORE), and one study reported both scores.

**Table 1:** Characteristics of included studies

Study	Country	Setting	Design	Sample size	Case	Follow‐up	Source of funding	Risk of bias (percentage)†
Fukuhara (2020)^15^	United States	Hospital	Case study	1	Aortic dissection repair	Short term (to clinic)	None	59.38
Fukuhara (2020)^16^	United States	Hospital	Case series	24	Aortic dissection repair	Hospital Admission	None	67.19
Hussain (2020)^17^	United Kingdom	Hospital	Case study	1	AVR	Hospital Admission	None	62.50
Rescigno (2020)^18^	United Kingdom	Hospital	Case study	1	CABG	Hospital Admission	None	62.50
Salna (2020)^19^	United States	Hospital	Case study	1	CABG	Hospital Admission	None	65.63
Silveira (2020)^20^	Brazil	Hospital	Case study	1	CABG	Hospital Admission	None	65.63
Varela Barca (2020)^21^	Spain	Hospital	Case study	1	AVR	Hospital Admission	None	59.38
Yandrapalli (2020)^22^	United States	Hospital	Case study	1	CABG	Hospital Admission	None	65.63
Farsky (2021)^23^	Brazil	Hospital	Case series	3	CABG	Hospital Admission	None	62.50
Romiti (2020)^24^	Italy	Hospital	Case study	1	CABG	Hospital Admission	None	62.50
Farina (2020)^25^	Italy	Hospital	Case study	1	CABG	Hospital Admission	None	60.94
Montandrau (2020)^26^	France	Hospital	Case study	1	CABG	Hospital Admission	None	64.06
Schwerzmann (2021)^27^	Switzerland	Hospital	Cohort	1	AVR + Ascending Aorta Replacement	Hospital Admission	Internal research funding	65.63
Soetisna (2021)^28^	Indonesia	Hospital	Case study	1	CABG	Hospital Admission	Not stated	64.06
Darvishi (2021)^29^	Iran	Hospital	Case study	1	CABG + Pericardiotomy	Hospital Admission	Not stated	56.25
Lopez‐Marco (2021)^30^	UK	Hospital	Cohort	3	Aortic Dissection Repair	Hospital Admission	None	64.06
Keaton‐Nasser (2021)^31^	USA	Hospital	Case study	1	CABG	Hospital Admission	Not stated	53.13
Omar(2021)^32^	Qatar	Hospital	Case Series	3	CABG	Hospital Admission	Corporate	62.50

† Average score on downs and black checklist.^28^

### Patient characteristics

Regarding patient characteristics in the overall cohort, 13 (29.5%) had type 2 diabetes mellitus, 29 (65.9%) had hypertension, 15 (34.1%) had a smoking history, three (6.8%) had chronic obstructive pulmonary disease COPD, two (4.5%) had preoperative stroke. The mean left ventricular ejection fraction (LVEF) was 46.8%, 10 (22.7%) had coronary artery disease and nine (20.5%) had previous cardiac surgery; of these, four (9.1%) had previous CABG surgery, and three (6.8%) had a past AVR.

### Patient outcomes

Across the included studies, 28 (63.6%) patients experienced postoperative complications. Of these, 12 (27.3%) experienced ARDS, six (13.6%) experienced cerebrovascular complications specifically and four (9.1%) patients required extracorporeal membrane oxygenation (ECMO). Both in‐hospital and 30‐day mortality was experienced in 12 (27.3%) patients. When reported, mean postoperative length of ICU stay was 7.4 days, and mean postoperative hospital length of stay was 14.5 days.

**Fig. 2:**
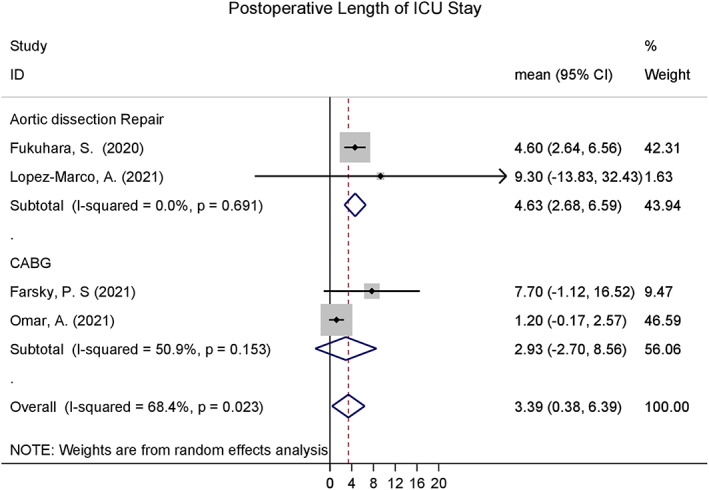
Postoperative length of ICU stay pooled across included studies.

**Fig. 3:**
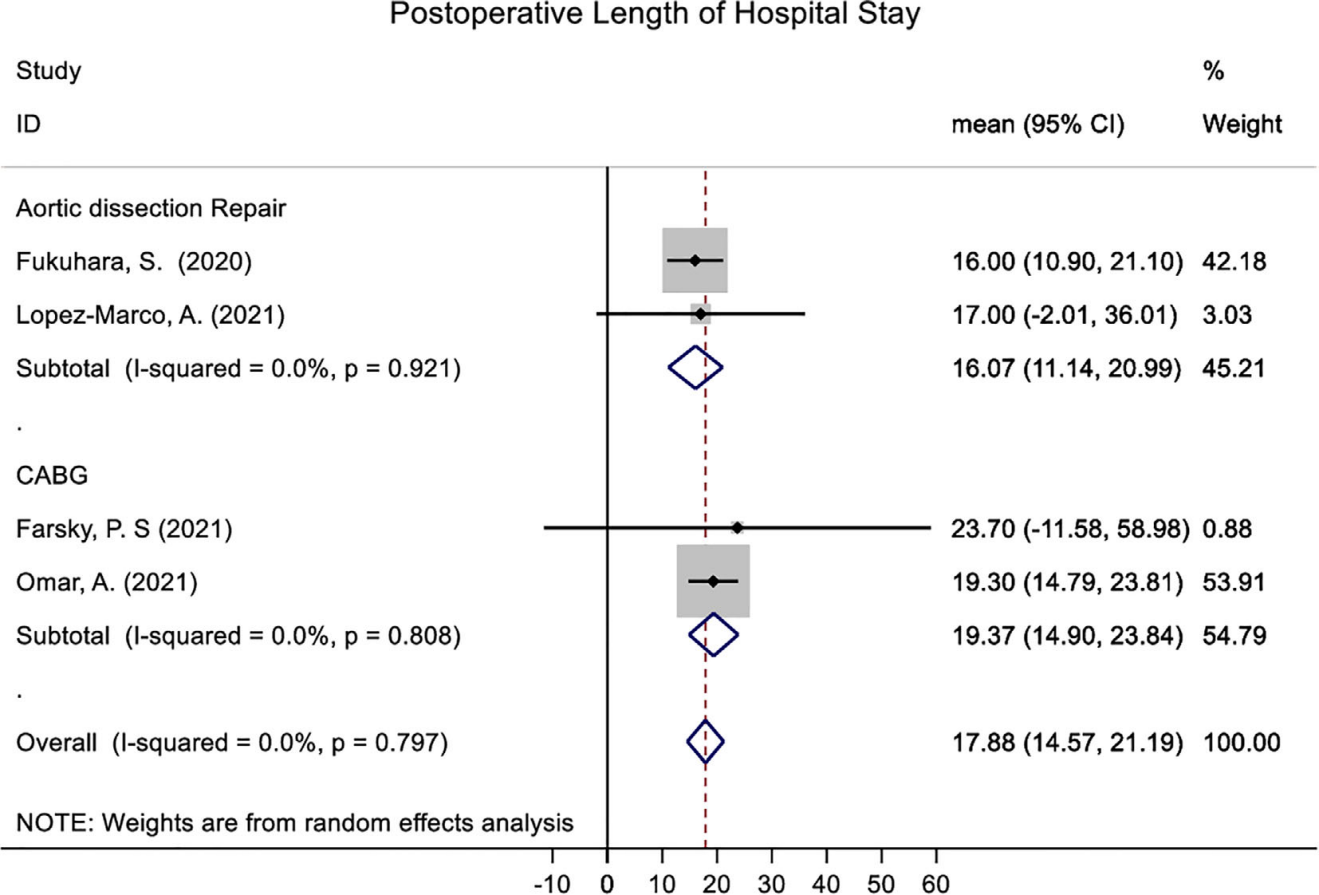
Postoperative length of hospital stay pooled across included studies.

Mean and standard deviation of Postoperative length of ICU and hospital stay was pooled across four studies using a random effects meta‐analysis model. Heterogeneity in the study estimates was assessed using the I‐squared statistic (0%) and Cochran's Q *P*‐value (0.797) which showed no heterogeneity. Because a random effects model was used degree of heterogeneity is not relevant however. The overall mean postoperative length of ICU stay was 3.39 (95% confidence interval (CI): 0.38, 6.39), while overall mean postoperative length of hospital stay was 17.88 (95% CI: 14.57, 21.19). This is shown in Figs 2 and 3. Meta‐analysis was not possible for the other planned outcomes.

### Risk of bias

The included studies were of moderate methodological quality upon critical appraisal using the Downs and Black checklist.^14^ Overall percentages on risk of bias assessments for the included studies can be found in Table 1 and the individual breakdown of these scores can be found in Data S4. Mean scores, representing the average of the two reviewer scores, were calculated for each category. Calculated means were as follows: total mean score 19.8 out of 32 (*SD* 1.22); reported sub‐scale mean score 6.1 out of 11 (*SD* 0.90); external validity sub‐scale mean score 3 out of 3 (*SD* 0); bias sub‐scale mean score 4.9 out of 7 (*SD* 0.16); confounding sub‐scale mean score 3.4 out of 6 (*SD* 0.36); power sub‐scale mean score 3.1 out of 5 (*SD* 0.51).

## Discussion

This systematic review and meta‐analysis is the first to characterize the global literature regarding outcomes after cardiac surgery in COVID‐19 positive patients. Our study suggests that COVID‐19 positive patients undergoing cardiac surgery have long postoperative length of stay in hospital and ICU and overall poor outcomes of high morbidity and mortality. Our review identified poor short‐term outcomes in patients undergoing cardiac surgery with concomitant COVID‐19 infection. Myocardial damage from infection may represent one large factor, combined with the physiological and inflammatory stresses associated with cardiopulmonary bypass, need for mechanical ventilation and ischaemia reperfusion.^35^ It is likely that the cohort studied represents cases of urgent and emergency surgery that could not otherwise be postponed, which has also been shown as a risk factor for poorer post‐operative outcomes.^36^, ^37^ Although not shown in our study, issues within hospitals and health systems may also contribute to this finding, as available resources may have been diverted to treat other COVID‐19 patients.

COVID‐19 infection causes significant damage to the cardiovascular system. A large meta‐analysis of 1527 patients found that 8% of patients with COVID‐19 had associated cardiac injury.^38^ There is a growing body of evidence suggesting that endothelial dysfunction through the Angiotensin‐converting enzyme 2 (ACE‐2) receptor can cause severe myocardial injury.^39^, ^40^, ^41^, ^42^ COVID‐19 uses severe acute respiratory syndrome coronavirus (SARS‐CoV) receptor ACE‐2 to enter host cells.^41^ It has been shown that susceptibility to SARS coronavirus S protein‐driven infection correlates with expression of ACE‐2,^43^ and accordingly, higher ACE‐2 expression may also lead to higher risk of SARS‐CoV‐2 infection. The systemic inflammatory response syndrome (SIRS) associated with COVID‐19, in severe disease, results in a cytokine release syndrome which can cause injury to vascular endothelium and cardiac myocytes.^44^, ^45^, ^46^, ^47^ A number of pro‐inflammatory cytokines are released, including interleukin‐2 (IL‐2), IL‐6, IL‐8, IL‐10 and tumour necrosis factor alpha (TNF‐α). During severe inflammation, this can result in asystemic inflammatory response syndrome (ARDS) and multi‐organ dysfunction.^48^, ^49^ The elevated cardiometabolic demand from systemic inflammation, combined with ongoing hypoxia caused by pneumonia or ARDS, is associated with myocardial damage.^46^ This process can also result in plaque rupture or coronary thrombosis causing acute coronary syndrome.^50^, ^51^ An American study of 21 patients admitted with COVID‐19 to an intensive care unit (ICU) demonstrated one‐third of patients had new‐onset cardiomyopathy with decreased left ventricular function and clinical signs of cardiogenic shock with elevated cardiac enzymes.^52^ In some case series, upto half of patients who died from COVID‐19 had some form of heart failure.^53^, ^54^

Arrhythmias and sudden cardiac arrest have been documented in patients with COVID‐19. These pathologies are thought to stem from cardiac injury arising from hypoxia, poor coronary perfusion, myocardial injury, SIRS and the proarrhythmic effect of certain medications used to treat COVID‐19. The pro‐inflammatory state brought on by COVID‐19 infection also has a significant role. Animal studies from past pandemics due to H1N1, SARS and Middle Eastern Respiratory Syndrome (MERS) showed a significant association between a past history of cardiovascular disease and myocardial injury due to cardiomyopathy causing atrioventricular dilatation and impaired ejection fraction.^55^ Severe COVID‐19 disease also manifests acute myocardial injury as a by‐product, demonstrated by the finding that patients who were admitted to ICU as a result of COVID‐19 infection had significantly higher creatine kinase myocardial band (CK‐MB) and troponin I levels.^39^ Elevated cardiac troponin levels is associated with ventricular tachycardia and fibrillation.^56^ Accordingly, the rate of developing cardiac arrhythmia was found to be more than six times greater for patients admitted to ICU. Given that many cardiovascular diseases are acute manifestations of underlying chronic disease processes, it is hypothesised that the hyper‐inflammatory microenvironment produced by COVID‐19 causes an imbalance between infection induced increase in metabolic demand and diminished cardiorespiratory reserve. This causes acute instability leading to coronary plaque events, arrhythmias and sudden cardiac death.^57^

The majority of evidence relating to cardiac surgery during COVID‐19 is opinion‐based, usually deriving from polls of cardiac surgeons across prominent centres. A poll of cardiac surgeons in the UK produced a consensus view that all patients should be screened for COVID‐19 pre‐surgery, full PPE at all times to prevent spread between patients and importantly the decision to hold a multidisciplinary team (MDT) meeting for every case of cardiac surgery in a COVID‐19 positive patient.^58^ These MDTs believed aortic and mitral surgery could be pursued in select cases however the role of CABG was more controversial amongst the surgeons. The CovidSurg Collaborative explored the impact of COVID‐19 on surgical services. Data from CovidSurg suggests that delaying surgery for 4 weeks after a positive COVID‐19 test is beneficial to outcomes in Cancer related surgeries, and this is echoed in the wider literature.^59^, ^60^ The CovidSurg Collaborative also found that swab testing all patients before major surgery was beneficial in preventing complications; this benefit was amplified in areas with higher COVID‐19 transmission and more radical surgeries.^59^ The most marked effect on surgical decision‐making has been the introduction of COVID‐19 screening for surgical patients. Initially this screening was CT based however more rapid swab kits are now the preferred method, these swab kits are more effective than CT screening alone.^59^

An important subgroup of patients who have been significantly affected by COVID‐19 are transplant recipients. These patients are routinely on immunosuppressive regimens to protect their grafts, regarding which there are limited data available, on immunosuppressive agents and their interactions with the COVID pathogen. Clearly, these are vulnerable patients. A multicentre review paper by Bottio et al.^61^ found a twofold increase in the prevalence of COVID‐19 amongst heart transplant recipients compared with the general population, and attributed this to the higher susceptibility to infections due to chronic immunosuppressive therapy. They noted that the 25% case fatality rate for heart transplant recipients, regardless of age, was equivalent to that of Italians aged over 70. There is some literature which has shown that ceasing immunosuppressive therapy can be associated with positive outcomes in COVID‐19 patients,^62^ however there is some contention, given that this can precipitate immunological memory and subsequent allograft rejection.

There are limitations to our study. Ten texts were identified through title and abstract screening which could not be retrieved, and given the small evidence base, the absence of these papers may impact our results. While our study aimed to include large cohort studies, more than half of included studies were single patient case reports which limits the generalizability of our results to larger, heterogeneous patient populations. Given that a large proportion of our studies originated from the USA and Western Europe, regional differences in the COVID‐19 virus and healthcare systems more broadly may not be accounted for. Further studies investigating the outcomes of COVID‐19 positive patients who undergo cardiac surgery may yield different results compared with our review, in particular more recent studies conducted after the date of our search. Our study did not capture COVID‐19 positive patients who required cardiac surgery but failed to survive in the operating room.^63^

During the COVID‐19 pandemic, cardiac surgery has faced many challenges. Many cardiac surgeons have had to change their daily practice, with some offering their services in critical care units to meet the demand caused by the pandemic.^64^ Not only have these placed surgeons into an unfamiliar environment, but the pandemic‐associated risks for healthcare workers contributes to an increased level of anxiety. Due to the redistribution of resources in many countries towards critical care services for patients suffering from COVID‐19, cardiac surgical procedures have been frequently delayed.^65^ As a result, there has been a significant decrease cardiac surgeries performed during the pandemic.^66^ Similarly, there has been a restructuring in the delivery of cardiac surgery to more centralized units.^67^ The combined effect of this has been to create a significant backlog of patients who require cardiac surgery but faced cancellations due to insufficient resources, hospital beds or personal protective equipment for staff.^68^ Patients who have a clinical urgency should be prioritized for treatment, a challenge given the ongoing pandemic setting.

## Conclusion

This systematic review and meta‐analysis investigated studies of limited quality which characterized outcomes after cardiac surgery in COVID‐19 positive patients. Our study demonstrates that these patients have poor outcomes. Further issues to be explored are the effect of COVID‐19 on decision‐making in cardiac surgery, and the effects of COVID‐19 on the cardiovascular system at a cellular level.

## Author contributions

**Aashray K. Gupta:** Conceptualization; data curation; formal analysis; investigation; methodology; project administration; validation; visualization; writing – original draft; writing – review and editing. **Alasdair Leslie:** Data curation; formal analysis; investigation; validation; writing – original draft; writing – review and editing. **Joseph N. Hewitt:** Data curation; formal analysis; investigation; methodology; visualization; writing – review and editing. **Joshua G. Kovoor:** Conceptualization; data curation; formal analysis; investigation; methodology; project administration; resources; validation; visualization; writing – original draft; writing – review and editing. **Christopher D. Ovenden:** Conceptualization; formal analysis; investigation; methodology; validation; visualization; writing – review and editing. **Suzanne Edwards:** Formal analysis; investigation; methodology; resources; software; visualization; writing – original draft; writing – review and editing. **Justin C. Y. Chan:** Formal analysis; investigation; methodology; supervision; validation; visualization; writing – review and editing. **Michael G. Worthington:** Formal analysis; investigation; methodology; supervision; writing – review and editing.

## Conflict of interest

None declared.

## Supporting information


**Appendix S1:** Supporting Information
