# Supplementary material for: Cardiac surgery on patients with COVID‐19: a systematic review and meta‐analysis
Source: ANZ J Surg. 2022 Apr 3;92(5):1007–14. doi: 10.1111/ans.17667 (PMC9111466; doi:10.1111/ans.17667)
Supplement: Supplementary file 1 — Appendix S1: Supporting Information [file ANS-92-1007-s001.docx]

**SUPPLEMENTARY DIGITAL CONTENT**

**List of Supplementary Digital Content**

- Appendix 1: PRISMA 2020 Checklist
- Appendix 2: MOOSE Checklist
- Appendix 3. Justification of exclusions at full-text review
- Appendix 4. Risk of bias assessment for included studies using the Downs and Black checklist

**Appendix 1 – PRISMA 2020 Checklist**

| **Section and Topic** | **Item #** | **Checklist item** | **Location where item is reported** |
| --- | --- | --- | --- |
| **TITLE** | | |  |
| Title | 1 | Identify the report as a systematic review. | Title page |
| **ABSTRACT** | | |  |
| Abstract | 2 | See the PRISMA 2020 for Abstracts checklist. | 2 |
| **INTRODUCTION** | | |  |
| Rationale | 3 | Describe the rationale for the review in the context of existing knowledge. | 3 |
| Objectives | 4 | Provide an explicit statement of the objective(s) or question(s) the review addresses. | 3 |
| **METHODS** | | |  |
| Eligibility criteria | 5 | Specify the inclusion and exclusion criteria for the review and how studies were grouped for the syntheses. | 4 |
| Information sources | 6 | Specify all databases, registers, websites, organisations, reference lists and other sources searched or consulted to identify studies. Specify the date when each source was last searched or consulted. | 4 |
| Search strategy | 7 | Present the full search strategies for all databases, registers and websites, including any filters and limits used. | 4 |
| Selection process | 8 | Specify the methods used to decide whether a study met the inclusion criteria of the review, including how many reviewers screened each record and each report retrieved, whether they worked independently, and if applicable, details of automation tools used in the process. | 4 |
| Data collection process | 9 | Specify the methods used to collect data from reports, including how many reviewers collected data from each report, whether they worked independently, any processes for obtaining or confirming data from study investigators, and if applicable, details of automation tools used in the process. | 4 |
| Data items | 10a | List and define all outcomes for which data were sought. Specify whether all results that were compatible with each outcome domain in each study were sought (e.g. for all measures, time points, analyses), and if not, the methods used to decide which results to collect. | 4 |
|  | 10b | List and define all other variables for which data were sought (e.g. participant and intervention characteristics, funding sources). Describe any assumptions made about any missing or unclear information. | 4 |
| Study risk of bias assessment | 11 | Specify the methods used to assess risk of bias in the included studies, including details of the tool(s) used, how many reviewers assessed each study and whether they worked independently, and if applicable, details of automation tools used in the process. | 4 |
| Effect measures | 12 | Specify for each outcome the effect measure(s) (e.g. risk ratio, mean difference) used in the synthesis or presentation of results. | 4 |
| Synthesis methods | 13a | Describe the processes used to decide which studies were eligible for each synthesis (e.g. tabulating the study intervention characteristics and comparing against the planned groups for each synthesis (item #5)). | 4 |
|  | 13b | Describe any methods required to prepare the data for presentation or synthesis, such as handling of missing summary statistics, or data conversions. | 4 |
|  | 13c | Describe any methods used to tabulate or visually display results of individual studies and syntheses. | 4 |
|  | 13d | Describe any methods used to synthesize results and provide a rationale for the choice(s). If meta-analysis was performed, describe the model(s), method(s) to identify the presence and extent of statistical heterogeneity, and software package(s) used. | 4 |
|  | 13e | Describe any methods used to explore possible causes of heterogeneity among study results (e.g. subgroup analysis, meta-regression). | 4 |
|  | 13f | Describe any sensitivity analyses conducted to assess robustness of the synthesized results. | 4 |
| Reporting bias assessment | 14 | Describe any methods used to assess risk of bias due to missing results in a synthesis (arising from reporting biases). | 4 |
| Certainty assessment | 15 | Describe any methods used to assess certainty (or confidence) in the body of evidence for an outcome. | 4 |
| **RESULTS** | | |  |
| Study selection | 16a | Describe the results of the search and selection process, from the number of records identified in the search to the number of studies included in the review, ideally using a flow diagram. | 5 |
|  | 16b | Cite studies that might appear to meet the inclusion criteria, but which were excluded, and explain why they were excluded. | 5, appendix |
| Study characteristics | 17 | Cite each included study and present its characteristics. | 5, Table 1 |
| Risk of bias in studies | 18 | Present assessments of risk of bias for each included study. | 6, Table 1, appendix |
| Results of individual studies | 19 | For all outcomes, present, for each study: (a) summary statistics for each group (where appropriate) and (b) an effect estimate and its precision (e.g. confidence/credible interval), ideally using structured tables or plots. | 5-6, figures 2-3, appendix |
| Results of syntheses | 20a | For each synthesis, briefly summarise the characteristics and risk of bias among contributing studies. | 5-6 |
|  | 20b | Present results of all statistical syntheses conducted. If meta-analysis was done, present for each the summary estimate and its precision (e.g. confidence/credible interval) and measures of statistical heterogeneity. If comparing groups, describe the direction of the effect. | 5-6, figures 2-3, appendix |
|  | 20c | Present results of all investigations of possible causes of heterogeneity among study results. | 5-6, figures 2-3, appendix |
|  | 20d | Present results of all sensitivity analyses conducted to assess the robustness of the synthesized results. | 5-6, figures 2-3, appendix |
| Reporting biases | 21 | Present assessments of risk of bias due to missing results (arising from reporting biases) for each synthesis assessed. | 5-6, figures, 2-3, appendix |
| Certainty of evidence | 22 | Present assessments of certainty (or confidence) in the body of evidence for each outcome assessed. | 8-11, figures 2-4, appendix |
| **DISCUSSION** | | |  |
| Discussion | 23a | Provide a general interpretation of the results in the context of other evidence. | 7-9 |
|  | 23b | Discuss any limitations of the evidence included in the review. | 7-9 |
|  | 23c | Discuss any limitations of the review processes used. | 7-9 |
|  | 23d | Discuss implications of the results for practice, policy, and future research. | 7-9 |
| **OTHER INFORMATION** | | |  |
| Registration and protocol | 24a | Provide registration information for the review, including register name and registration number, or state that the review was not registered. | 4 |
|  | 24b | Indicate where the review protocol can be accessed, or state that a protocol was not prepared. | 4 |
|  | 24c | Describe and explain any amendments to information provided at registration or in the protocol. | Not applicable |
| Support | 25 | Describe sources of financial or non-financial support for the review, and the role of the funders or sponsors in the review. | Title page |
| Competing interests | 26 | Declare any competing interests of review authors. | Title page |
| Availability of data, code and other materials | 27 | Report which of the following are publicly available and where they can be found: template data collection forms; data extracted from included studies; data used for all analyses; analytic code; any other materials used in the review. | Title page |

**Appendix 2 – MOOSE Checklist**

| **Reporting Criteria** | **Reported (Yes/No)** | **Reported on Page Number** |
| --- | --- | --- |
| Problem definition | Yes | 3 |
| Hypothesis statement | Yes | 3 |
| Description of study outcomes | Yes | 4 |
| Type of exposure or intervention used | Yes | 4 |
| Type of study designs used | Yes | 4 |
| Study population | Yes | 4 |
| Qualifications of searchers (e.g. librarians and investigators) | No | - |
| Search strategy, including time period included in the synthesis and keywords | Yes | 4, Appendix |
| Effort to include all available studies, including contact with authors | Yes | 4 |
| Databases and registries searched | Yes | 4 |
| Search software used, name and version, including special features used (e.g. explosion) | Yes | 4 |
| Use of hand searching (e.g. reference lists of obtained articles) | Yes | 4 |
| List of citations located and those excluded, including justification | Yes | 4, Appendix |
| Methods of addressing articles published in languages other than English | Yes | 4 |
| Method of handling abstracts and unpublished studies | Yes | 4 |
| Description of any contact with authors | Yes | No contact with authors |
| Description of relevance or appropriateness of studies assembled for assessing the hypothesis to be tested | Yes | 4 |
| Rationale for the selection and coding of data (e.g. sound clinical principles or convenience) | Yes | 4 |
| Documentation of how data were classified and coded (e.g. multiple raters, blinding, and interrater reliability) | Yes | 4 |
| Assessment of confounding (e.g. comparability of cases and controls in studies where appropriate) | Yes | 5-6 |
| Assessment of study quality, including blinding of quality assessors; stratification or regression on possible predictors of study results | Yes | 5-6 |
| Assessment of heterogeneity | Yes | 5-6 |
| Description of statistical methods (e.g. complete description of fixed or random effects models, justification of whether the chosen models account for predictors of study results, dose-response models, or cumulative meta-analysis) in sufficient detail to be replicated | Yes | 4-6 |
| Provision of appropriate tables and graphics | Yes | Throughout manuscript |
| Graphic summarizing individual study estimates and overall estimate | Yes | Figures 2–3, appendix |
| Table giving descriptive information for each study included | Yes | Table 1 |
| Results of sensitivity testing (e.g. subgroup analysis) | Yes | 5-6 |
| Indication of statistical uncertainty of findings | Yes | 4-9 |
| Quantitative assessment of bias (e.g. publication bias) | Yes | 4-9 |
| Justification for exclusion (e.g. exclusion of non-English-language citations) | Yes | 4-5, Figure 1, Appendix |
| Assessment of quality of included studies | Yes | 5-6, Table 1, Appendix |
| Consideration of alternative explanations for observed results | Yes | 7-10 |
| Generalization of the conclusions (i.e. appropriate for the data presented and within the domain of the literature review) | Yes | 7-10 |
| Guidelines for future research | Yes | 7-10 |
| Disclosure of funding source | Yes | Title page |

**Appendix 3 – Justification of exclusions at full-text review**

**Studies included at full-text review**

1. Fukuhara, S. and Rosati, C. M. and El-Dalati, S. Acute Type A Aortic Dissection During the COVID-19 Outbreak
2. Fukuhara, S. and Tang, H. and Kim, K. M. and Tan, L. and Shen, K. and Song, G. and Tang, T. and Patel, H. J. and Wei, X. and Yang, B. Type A Aortic Dissection During COVID-19 Pandemic: Report From Tertiary Aortic Centers in the United States and China
3. Hussain, A. and Khan, H. and Lopez-Marco, A. and Roberts, N. and Oo, A. Cardiac surgery in patients with confirmed COVID-19 infection: Early experience
4. Rescigno, G. and Firstenberg, M. and Rudez, I. and Uddin, M. and Nagarajan, K. and Nikolaidis, N. A Case of Postoperative Covid-19 Infection After Cardiac Surgery: Lessons Learned.
5. Salna, M. and Polanco, A. and Bapat, V. and George, I. and Argenziano, M. and Takeda, K. A case of coronavirus disease 2019 (COVID-19) presenting after coronary artery bypass grafting
6. Silveira, Lucas Molinari Veloso da and Guerreiro, Gustavo Pampolha and Lisboa, Luiz Augusto Ferreira and Mejia, Omar Asdrubal Vilca and Dallan, Luis Roberto Palma and Dallan, Luis Alberto Oliveira and Jatene, Fabio B. Coronary Artery Bypass Graft During the COVID-19 Pandemic
7. Varela Barca, Laura and Torralba Cloquell, Isabel and Herrero Cereceda, Jaime and Saez de Ibarra, Jose Ignacio. An unexplained death after routine cardiac surgery: how long have we dealt with coronavirus disease 2019?
8. Yandrapalli, S. and Cooper, H. A. and Malekan, R. Successful coronary artery bypass operation in a SARS-COV-2 infected patient with acute coronary syndrome.
9. Farsky, P. S. and Feriani, D. and Valente, B. B. P. and Andrade, M. A. G. and Amato, V. L. and Carvalho, L. and Ibanes, A. S. and Godoy, L. Coronary Artery Bypass Surgery in Patients With COVID-19: What Have We Learned?
10. Romiti, S. and Totaro, M. and Laderchi, A. and Peruzzi, M. and Vinciguerra, M. and Greco, E. Case Report: Emergency CABG Following Failure of PTCA in a COVID-19 Patient
11. Farina, A. and Uccello, G. and Spreafico, M. and Bassanelli, G. and Savonitto, S. SARS-CoV-2 detection in the pericardial fluid of a patient with cardiac tamponade.
12. Montrandrau, O. and Arana, H. and Ehooman, F. and Bouattour, K. and Cherruault, M. and Ouechani, W. and Hamdaoui, I. and Dufour, G. and Kattou, F. and Philip, I. and Beaussier, M. Surgical Revascularization With Cardiopulmonary Bypass on a Patient With Severe COVID-19
13. Clinical outcome of COVID-19 in patients with adult congenital heart diseaseSchwerzmann, M. and Ruperti-Repilado, F. J. and Baumgartner, H. and Bouma, B. and Bouchardy, J. and Budts, W. and Campens, L. and Chessa, M. and Del Cerro Marin, M. J. and Gabriel, H. and Gallego, P. and Garcia-Orta, R. and Gonzalez, A. E. and Jensen, A. S. and Ladouceur, M. and Mir and a-Barrio, B. and Morissens, M. and Pasquet, A. and Rueda, J. and van den Bosch, A. E. and van der Zwaan, H. B. and Tobler, D. and Greutmann, M.
14. A 48-Year-Old Man at Low Risk for SARS-CoV-2 Infection Who Underwent Planned Elective Triple-Vessel Coronary Artery Bypass Graft Surgery at a National Heart Center in Indonesia Followed by a Fatal Case of COVID-19 Soetisna, T. W. and Buana, A. C. and Tirta, E. S. and Ardiyan, A. and Aligheri, D. and Herlambang, B. and Tjub and i, A. and Hanafy, D. A. and Sugisman, S.
15. Darvishi M, Shahali H. Acute Cardiac Tamponade: A Case of Life-Threatening Coronavirus Disease 2019 Complication During Air Medical Transportation. Air Med J. 2021 May-Jun;40(3):179-181.
16. Lopez-Marco A, Harky A, Malvindi PG, Verdichizzo D, McPherson I, Roman M, Oo A, Ohri S. Type A aortic syndromes in COVID-19 positive patients: Case series from a UK multicentre study. J Card Surg. 2021 Aug;36(8):2692-2696.
17. [Keaton Nasser](https://www.jacc.org/doi/10.1016/S0735-1097%2821%2903384-2), [Jibran Rana](https://www.jacc.org/doi/10.1016/S0735-1097%2821%2903384-2), [Chowdhury Ahsan](https://www.jacc.org/doi/10.1016/S0735-1097%2821%2903384-2). COVID-19 AND ACUTE CORONARY SYNDROME WITH MULTI-VESSEL DISEASE NEEDING CABG. [J Am Coll Cardiol](https://www.jacc.org/journal/jacc). 2021 May, 77 (18_Supplement_1) 2028
18. Omar AS, Shoman B, Sudarsanan S, Shouman Y. Chest radiography requirements for patients with asymptomatic COVID-19 undergoing coronary artery bypass surgery: Three case reports. World J Virol. 2021 May 25;10(3):130-136.

**Studies excluded at full-text review**

**Wrong population**

1. Sromicki, J. and Schmiady, M. and Maisano, F. and Mestres, C. A. ECMO therapy in COVID-19: The Zurich experience
2. Harky, A. and Harrington, D. and Nawaytou, O. and Othman, A. and Fowler, C. and Owens, G. and Torella, F. and Kuduvalli, M. and Field, M. COVID-19 and cardiac surgery: A perspective from United Kingdom
3. Challenges in heart transplantation during COVID-19: A single-center experience Singhvi, A. and Barghash, M. and Lala-Trindade, A. and Mitter, S. S. and Parikh, A. and Oliveros, E. and Rollins, B. M. and Brunjes, D. L.
4. Management of acute type A aortic dissection during COVID-19 outbreak: Experience from AnzhenZhang, C. H. and Ma, W. G. and Zhong, Y. L. and Ge, Y. P. and Li, C. N. and Qiao, Z. Y. and Liu, Y. M. and Zhu, J. M. and Sun, L. Z.
5. COVID-19 in patients recovering from cardiac surgery: A surprising mild disease course Cerillo, A. G. and Marchionni, N. and Bacchi, B. and Stefano, P.
6. Transcatheter aortic valve replacement during the COVID-19 pandemic-A Dutch single-center analysis Rooijakkers, Maxim J. P. and Li, Wilson W. L. and Wollersheim, Laurens W. L. M. and Geuzebroek, Guillaume S. C. and Gehlmann, Helmut and van Garsse, Leen A. F. M. and van Wely, Marleen H. and Verkroost, Michel W. A. and Morshuis, Wim J. and Wertheim, Heiman and van Royen, Niels
7. A case series of novel coronavirus infection in heart transplantation from 2 centers in the pandemic area in the North of Italy Iacovoni, A. and Boffini, M. and Pidello, S. and Simonato, E. and Barbero, C. and Sebastiani, R. and Vittori, C. and Fontana, A. and Terzi, A. and De Ferrari, G. M. and Rinaldi, M.
8. Peri-operative COVID-19 infection in urgent elective surgery during a pandemic surge period: a retrospective observational cohort study Kane, A. D. and Paterson, J. and Pokhrel, S. and Berry, S. K. and Monkhouse, D. and Br and , J. W. and Ingram, M. and Danjoux, G. R.
9. Initial Report From a Swedish High-volume Transplant Center After the First Wave of the COVID-19 Pandemic Felldin, M. and Søftel and , J. M. and Magnusson, J. and Ekberg, J. and Karason, K. and Schult, A. and Larsson, H. and Oltean, M. and Friman, V.
10. Performing an urgent neonatal cardiac intervention safely during the COVID-19 pandemic Spencer, R. and Chaves, D. V. and Brooks, M. C. and Goldshtrom, N. and Moroz, L. and Miller, R. and Glickstein, J. and Levasseur, S. and Bacha, E. A. and Turner, M. E. and Farooqi, K. M.
11. Giamberti, Aless and ro and Varrica, Aless and ro and Agati, Salvatore and Gargiulo, Gaetano and Luciani, Giovanni Battista and Marianeschi, Stefano Maria and Pace Napoleone, Carlo and Oppido, Guido and Brunelli, Federico and Palma, Gaetano and Pak, Vitali and Arcieri, Luigi and Scalzo, Gabriele and Padalino, Massimo and Galletti, Lorenzo and Congenital Domain of the Italian Soc Impact of the coronavirus disease 2019 (COVID-19) pandemic on the Italian congenital cardiac surgery system: a national survey
12. Hu, X. J. and Wang, Y. and Xia, J. H. and Dong, N. G. and Shi, J. W. Managements of 13 emergency cardiac surgeries under COVID-19 pandemic in a Sentinel Hospital
13. Inama, G. and Dodi, C. and Provini, M. and Bossoni, E. and Inama, L. and Balzarini, L. and Mancini, C. and Ramponi, S. and Marvisi, M.Coronavirus disease 2019 infection in patients with recent cardiac surgery: does chronic anticoagulant therapy have a protective effect?
14. Ambulatory TAVR: Early Feasibility Experience During the COVID-19 Pandemic Zouaghi, O. and Wintzer-Wehekind, J. and Lienhart, Y. and Abdellaoui, M. and Faurie, B.
15. Turn down of acute aortic syndrome cases during COVID-19: Results from UK multicentre studies Roman, M. and Harky, A. and Brazier, A. and Lim, K. and Gradinariu, G. and Oo, A. and Mariscalco, G. and Lopez-Marco, A
16. Severe parapharyngeal abscess that developed significant complications: management during the COVID-19 pandemic Ajeigbe, T. and Ria, B. and Wates, E. and Mattine, S.
17. A cohort study of 30 day mortality after NON-EMERGENCY surgery in a COVID-19 cold site Kasivisvanathan, V. and Lindsay, J. and Rakshani-Moghadam, S. and Elhamshary, A. and Kapriniotis, K. and Kazantzis, G. and Syed, B. and Hines, J. and Bex, A. and Ho, D. H. and Hayward, M. and Bhan, C. and MacDonald, N. and Clarke, S. and Walker, D. and Bellingan, G. and Moore, J. and Rohn, J. and Muneer, A. and Roberts, L. and Haddad, F. and Kelly, J. D.
18. Ventricular septal defect complicating delayed presentation of acute myocardial infarction during COVID-19 lockdown: a case report Evans, M. C. and Steinberg, D. H. and Rhodes, J. F. and Tedford, R. J.
19. Thoracic Surgery during Coronavirus Disease 2019 (COVID-19): The Experience of a Level 1 Trauma Center Smelt, J. and Santhirakumaran, G. and Vaughan, P. and Hunt, I. and Tan, C. R.
20. Cinar, G. and Sancaoglu, C. and Inan, B. and Dincer, I. and Cakici, M. and Sayin, T. and Azap, A. and Akar, A. R. Heart transplant recipient survivor from COVID-19: The first case of Turkey
21. Doglietto, F. and Vezzoli, M. and Gheza, F. and Lussardi, G. L. and Domenicucci, M. and Vecchiarelli, L. and Zanin, L. and Saraceno, G. and Signorini, L. and Panciani, P. P. and Cast Factors Associated With Surgical Mortality and Complications Among Patients With and Without Coronavirus Disease 2019 (COVID-19) in Italy
22. Gonzalez-Calatayud, M. and Vargas-Abrego, B. and Gutierrez-Uvalle, G. E. and Lopez-Romero, S. C. and Gonzalez-Perez, L. G. and Carranco-Martinez, J. A. and Raful-Zacarias-Ezzat, J. and Grac Observational study of the suspected or confirmed cases of sars COV-2 infection needing emergency surgical intervention during the first months of the pandemic in a third level hospital: Case se
23. Holzhauser, L. and Lourenco, L. and Sarswat, N. and Kim, G. and Chung, B. and Nguyen, A. B.Early experience of COVID-19 in 2 heart transplant recipients: Case reports and review of treatment options
24. Hsu, J. J. and Gaynor, P. and Kamath, M. and Fan, A. and Al-Saffar, F. and Cruz, D. and Nsair, A. COVID-19 in a high-risk dual heart and kidney transplant recipient
25. Hussain, A. and Roberts, N. and Oo, A. Prosthetic aortic valve endocarditis complicated by COVID-19 and haemorrhage
26. Prosthetic heart valves and the COVID-19 pandemic era: What should we be concerned about? Omidi, N. and Forouzannia, S. K. and Poorhosseini, H. and Tafti, S. H. A. and Salehbeigi, S. and Lotfi-Tokaldany, M.
27. Congenital cardiac interventions during the peak phase of COVID-19 pandemics in the country in a pandemics hospital in Istanbul Ugurlucan, M. and Yildiz, Y. and Oztas, D. M. and Coban, S. and Beyaz, M. O. and Sari, G. and Ulukan, M. O. and Karakaya, A. and
28. Surgical interventions in patients hospitalised with COVID-19. A review of seven months of experience working in a COVID-19 dedicated Rymarowicz, J. and Pędziwiatr, M. and Major, P. and Donohue, B. and Ciszek, K. and Nowakowski, M.
29. COVID-19 in an Adult With Tricuspid Atresia S/P Fontan Palliation Ahluwalia, N. and Love, B. and Chan, A. and Zaidi, A. N.
30. The role of concomitant cardiovascular diseases and cardiac biomarkers for predicting mortality in critical COVID-19 patients Aladağ, N. and Atabey, R. D.
31. Acute Myocardial Infarction and Papillary Muscle Rupture in the COVID-19 Era Aslam, S. and Mehra, M. R.
32. Regional Survey in Lombardy, Northern Italy, on Vascular Surgery Intervention Outcomes During The COVID-19 Pandemic Bellosta, R. and Piffaretti, G. and Bonardelli, S. and Castelli, P. and Chiesa, R. and Frigerio, D. and Lanza, G. and Pirrelli, S. and Rossi, G. and Trimarchi, S. and Briolini, F. and Cefali, P. and Caronno, R. and Arzini, A. and Diaco, D. and Baratta, V. and Aiello, S. and Molinari, A. C. L. and Giovannini, F. and Socrate, A. M. and Ferraris, M. and Silvestro, A. and Canu, G. and Costantini, E. and Logaldo, D. and Romani, F. and Lista, A. and Busoni, C. and Setti, M. and Mezzetti, R. and Sala, P. and Bassi, L. and Luzzani, L. and Pegorer, M. A. and Attisani, L. and Carugati, C. and Vescovi, M. and Trabattoni, P. and Zoli, S. and Rignano, A. and Magri, C. and V and one, P. and Losa, S. and Civilini, E. and Nano, G. and Mazzaccaro, D. and Tolva, V. and Lanza, J. and Curci, R. and Simonetti, G. and Lomazzi, C. and Grassi, V. and Bissacco, D. and Kahlberg, A. and Mascia, D. and Dallatana, R. and Carmo, M. and Ragni, F. and Marone, E. M. and Bozzani, A. and Tozzi, M. and Franchin, M. and Lussardi, G. and Segramora, V. and Deleo, G. and Crippa, M. and Porretta, T. and Viani, M. and Stegher, S. and Foresti, D. and Bonalumi, G.
33. Vascular Surgery During COVID-19 Emergency in Hub Hospitals of Lombardy: Experience on 305 Patients Kahlberg, A. and Mascia, D. and Bellosta, R. and Attisani, L. and Pegorer, M. and Socrate, A. M. and Ferraris, M. and Trabattoni, P. and Rinaldi, E. and Melloni, A. and Monaco, F. and Melissano, G. and Chiesa, R.
34. Surgery and COVID-19: Balancing the nosocomial risk a french academic center experience during the epidemic peak Ingels, A. and Bibas, S. and Da Costa, J. B. and Attias, A. and Brunetti, F. and De Angelis, N. and Desgranges, P. and Flouzat-Lachaniette, C. H. and Folliguet, T. and Ivanov, T. and Langeron, O. and Lelde, L. and Levesque, E. and Corvoisier, P. L. and Marmorat, C. and Melendugno, F. and Meningaud, J. P. and Mesli, F. and Paillusson, W. and Palfi, S. and Pedre, L. and Somacale, D. and Champy, C. M. and de la Taille, A.
35. Outcomes of Vascular and Endovascular Interventions Performed During the Coronavirus Disease 2019 (COVID-19) Pandemic: The Vascular and Endovascular Research Network (VERN) Covid-19 Vascular Service (COVER) Tier 2 Study Benson, R. A. and N and hra, S.
36. Acute Increase in Deaths Among Patients With Adult Congenital Heart Disease During COVID-19: Single-Center Experience Dawson, K. L. and Vincent, L. L. and Krieger, E. V. and Stout, K. K. and Buber, J.
37. Clinical characteristics and outcomes of patients undergoing surgeries during the incubation period of COVID-19 infection Lei, S. and Jiang, F. and Su, W. and Chen, C. and Chen, J. and Mei, W. and Zhan, L. Y. and Jia, Y. and Zhang, L. and Liu, D. and Xia, Z. Y. and Xia, Z.
38. Single-Center Vascular Hub Experience after 7 weeks of COVID-19 Pandemic in Lombardy (Italy) Mascia, D. and Kahlberg, A. and Melloni, A. and Rinaldi, E. and Melissano, G. and Chiesa, R.
39. Non-Elective Pediatric Cardiac Catheterization During COVID-19 Pandemic: A New York Center Experience Oshiro, K. T. and Turner, M. E. and Torres, A. J. and Crystal, M. A. and Vincent, J. A. and Barry, O. M.
40. Lessons for Emergency Surgery in the Second Wave: One-Month Single-Centre Experience During the First Wave of COVID-19Shakir, T. and Iqbal, M. R. and Darwish, N. M. and Kirmani, N.
41. Characteristics and Outcomes of Patients With a Left Ventricular Assist Device With Coronavirus Disease-19 Sobol, I. and Yuzefpolskaya, M. and Roth, Z. and Colombo, P. C. and Horn, E. and Takeda, K. and Sayer, G. and Uriel, N. and Naka, Y.
42. Ventricular septal rupture and cardiogenic shock complicating STEMI during COVID-19 pandemic: An old foe re-emergesTehrani, B. and Batchelor, W.
43. Mortality in STEMI Patients During the COVID Era: Has the Pandemic Changed Our Clinical Practice? Barbieri, L. and Tumminello, G. and Lucreziotti, S. and Gentile, D. and Centola, M. and Conconi, B. and Carlà, M. and Mafrici, A. and Carugo, S.
44. Differences in hub and spoke vascular units practice during the novel Coronavirus-19 (COVID-19) outbreak in Lombardy, Italy Bellosta, R. and Bissacco, D. and Rossi, G. and Pirrelli, S. and Lanza, G. and Frigerio, D. and Chiesa, R. and Castelli, P. and Bonardelli, S. and Trimarchi, S.
45. Testing the Asymptomatic Pre-Surgical Population for Severe Acute Respiratory Syndrome Coronavirus 2 Bobenchik, A. M. and Maslow, A. D. and Angus, A. B. and Murphy, J. and Kurtis, J. D. and Chapin, K. C.
46. Severe Aortic Thrombosis in the Early Period after COVID-19: Two Cases Borulu, F. and Erkut, B.
47. Endovascular Surgery during COVID-19 Virus Pandemic as a Valid Alternative to Open Surgery Bozzani, A. and Arici, V. and Ticozzelli, G. and Tavazzi, G. and Sterpetti, A. V. and Mojoli, F. and Bruno, R. and Ragni, F.
48. Outcomes After Vascular Surgery Procedures in Patients with COVID-19 Infection: A National Multicenter Cohort Study (COVID-VAS) Outcomes After Vascular Surgery Procedures in Patients with COVID-19 Infection: A National Multicenter Cohort Study (COVID-VAS)
49. Akil, A. and Muller, M. C. and Ziegeler, S. and Dickgreber, N. and Fischer, S. Successful treatment of a geriatric COVID-19 patient with severe chest trauma An interdisciplinary case report
50. Acute Arterial Thromboembolism in Patients with COVID-19 in the New York City Area Etkin, Y. and Conway, A. M. and Silpe, J. and Qato, K. and Carroccio, A. and Manvar-Singh, P. and Giangola, G. and Deitch, J. S. and Davila-Santini, L. and Schor, J. A. and Singh, K. and Mussa, F. F. and L and is, G. S.
51. Thoracic surgery outcomes for patients with Coronavirus Disease 2019 Chang, S. H. and Chen, D. and Paone, D. and Geraci, T. C. and Scheinerman, J. and Bizekis, C. and Zervos, M. and Cerfolio, R. J.
52. Postoperative COVID-19 Pneumonia following Resection of a Large Thoracic Chondrosarcoma Kaidi, A. C. and Held, M. B. and Park, P. J. and Tyler, W. K.
53. Should We Postpone Elective Cardiovascular Procedures and Percutaneous Coronary Interventions During the COVID-19 Pandemic? Keskin, G. and Khalil, E. and Uysal, A.
54. Fattouch, K. and Corrao, S. and Augugliaro, E. and Minacapelli, A. and Nogara, A. and Zambelli, G. and Argano, C. and Moscarelli, M. Cardiac surgery outcomes in patients with coronavirus disease 2019 (COVID-19): A case-series report
55. Lopez-Marco, A. and Harky, A. and Verdichizzo, D. and Hope, E. and Rosser, B. and McPherson, I. and Kelly, R. and Holl and , L. and Ye Oo. Early experience of aortic surgery during the COVID-19 pandemic in the UK: A multicentre study.
56. Katsiampoura, A. and Perozo, C. and Varkaris, A. and Vellayappan, S. and Tam, M. Z. and Vellayappan, U. and Agnihotri, A. and Tam, S. Covid-19 positivity affects outcome of cardiac surgical patients.
57. Sanders, J. and Akowuah, E. and Cooper, J. and Kirmani, B. H. and Kanani, M. and Acharya, M. and Jeganathan, R. and Krasopoulos, G. and Ngaage, D. and Deglurkar, I. and Yiu, P. and Kendall, S. and Oo, A. Y. Cardiac surgery outcome during the COVID-19 pandemic: a retrospective review of the early experience in nine UK centres.
58. COVIDSurg Collaborative. Machine learning risk prediction of mortality for patients undergoing surgery with perioperative SARS-CoV-2: the COVIDSurg mortality score. Br J Surg. 2021 Nov 11;108(11):1274-1292.
59. Abdalghafoor T, Sarhan H, Kindawi A. Mitral valve replacement through mini sternotomy after long ECMO course: Case report. Perfusion. 2021 Mar 29:2676591211003237
60. Al Masri E, Redwan B, Thiel B, Ellger B, Begher C, Biancosino C, Kösek V. Clinical Outcome in Patients with Nosocomial COVID-19 Infection After Thoracic Surgery. Adv Exp Med Biol. 2021 Sep 23.
61. Aljehani Y, Othman SA, Almubarak Y, Elbaz A, Sabry M, Alreshaid F, Elbawab HY, Alghamdi ZM, Alshahrani M. Thoracic Surgery Consultations in COVID-19 Critically Ill Patients: Beyond Conservative Approach. Crit Care Res Pract. 2021 Mar 27;2021:6626150.
62. Belfort DSP, Biselli B, Ávila MS, Lira MTSS, Galas FRBG, Steffen SP, Gaiotto FA, Jatene FB, Bocchi EA, Ferreira SMA. COVID-19 complicating perioperative management of LVAD implantation: A case report and systematic review. J Card Surg. 2021 Sep;36(9):3405-3409.
63. Briani M, Torracca L, Crescenzi G, Barbone A. Impella 5.0 support before, during, and after surgical ventriculoplasty following acute myocardial infarction in the COVID-19 era: a case report. Eur Heart J Case Rep. 2021 Mar 7;5(3):ytab037
64. Chang SH, Chen D, Paone D, Geraci TC, Scheinerman J, Bizekis C, Zervos M, Cerfolio RJ. Thoracic surgery outcomes for patients with Coronavirus Disease 2019. J Thorac Cardiovasc Surg. 2021 Dec;162(6):1654-1664.
65. Day E, Fiorentino F, Abdelkhalek M, Smail H, Stock UA, Bhudia S, De Robertis F, Bahrami T, Raja S, Gaer J. The results of cardiac surgery during the COVID-19 pandemic compared with previous years: a propensity weighted study of outcomes at six months. J R Soc Med. 2022 Feb 7:1410768221077357.
66. Fattouch K, Corrao S, Augugliaro E, Minacapelli A, Nogara A, Zambelli G, Argano C, Moscarelli M. Cardiac surgery outcomes in patients with coronavirus disease 2019 (COVID-19): A case-series report. J Thorac Cardiovasc Surg. 2022 Mar;163(3):1085-1092.e3
67. Gaudriot B, Mansour A, Thibault V, Lederlin M, Cauchois A, Lelong B, Ross JT, Leurent G, Tadié JM, Revest M, Verhoye JP, Flecher E, Nesseler N. Successful heart transplantation for COVID-19-associated post-infectious fulminant myocarditis. ESC Heart Fail. 2021 Aug;8(4):2625-2630.
68. Giacomin E, Barioli A, Favero L, Lanzellotti D, Calzolari D, Daniotti A, Cernetti C. Safety and Feasibility of Transcatheter Aortic Valve Replacement in COVID-19 Patients: A Case Series. Cardiovasc Revasc Med. 2021 Jul;28S:68-71
69. Gidea, C.G. & Moazami, N. & Neumann, H. & Fargnoli, A. & Pavone, J. & Lewis, T. & Saraon, Tajinderpal & Goldberg, R. & Kadosh, Bernard & Katz, S. & Rao, Sundaresh & Metha, S. & Smith, Deane & Reyentovich, A.. (2021). COVID-19 Impact on Heart Organ Transplantation - New Insights from a Single-Center Experience. The Journal of Heart and Lung Transplantation. 40. S20.
70. Gopal K, Ahamed H, Jose R, Varma PK. Cardiac tamponade as a presentation of COVID-19 after cardiac surgery. Indian J Thorac Cardiovasc Surg. 2021 Nov 23:1-4.
71. Hernández MTG, Barreda CF, Valentín NMN, Gago MGF, López MFJ. CARACTERÍSTICAS CLÍNICAS Y RESULTADOS POSTOPERATORIOS DE LOS PACIENTES CON ANTECEDENTES DE ENFERMEDAD POR CORONAVIRUS (COVID-19) SOMETIDOS A INTERVENCIONES QUIRÚRGICAS TORÁCICAS [CLINICAL FEATURES AND POSTOPERATIVE OUTCOMES OF PATIENTS UNDERGOING THORACIC SURGERY AFTER SARS-COV-2 INFECTION]. Cir Esp. 2021 Oct 25. Spanish.
72. Hu L, Gao L, Zhang D, Hou Y, He LL, Zhang H, Liang Y, Xu J, Chen C. The incidence, risk factors and outcomes of acute kidney injury in critically ill patients undergoing emergency surgery: a prospective observational study. BMC Nephrol. 2022 Jan 22;23(1):42.
73. Hussain A, Khalil A, Kolvekar P, Gupta P, Kolvekar S. COVID-19 related mortality in post-operative cardiac surgical patients. J Cardiothorac Surg. 2021 Apr 26;16(1):112.
74. Isik M, Yıldırım S, Dereli Y, Tanyeli O, Görmüş N. Management of Cardiac Surgery in a Pandemic Region Hospital: Precautions, Results and Experiences During COVID-19. Eurasian J Med. 2021 Oct;53(3):208-213.
75. Keskin G, Uysal A, Erturk E, Hafız E, Dogan OF. Urgent Percutaneous Coronary Artery Intervention and Coronary Artery Bypass Grafting in STEMI Patients with Confirmed COVID-19. Heart Surg Forum. 2021 Jun 23;24(3):E564-E574.
76. Khalil KH, Sá MPBO, Vervoort D, Roever L, Pires MAA, Lima JMO, de Salles FB, Khalil GM, Nicz PFG, Mejía OAV, Okino AA, Lima RC. Impact of the COVID-19 pandemic on coronary artery bypass graft surgery in Brazil: A nationwide perspective. J Card Surg. 2021 Sep;36(9):3289-3293.
77. Kite TA, Ladwiniec A, Owens CG, Chase A, Shaukat A, Mozid AM, O'Kane P, Routledge H, Perera D, Jain AK, Palmer N, Hoole SP, Egred M, Sinha MK, Cahill TJ, Candilio L, Anantharam B, Byrne J, Walsh SJ, McEntegart M, Kean S, Siddique L, Budgeon C, Curzen N, Berry C, Ludman P, Gershlick AH; UK-ReVasc Registry Investigators. Outcomes following PCI in CABG candidates during the COVID-19 pandemic: The prospective multicentre UK-ReVasc registry. Catheter Cardiovasc Interv. 2021 May 4:10.1002/ccd.29702.
78. Kothari AN, DiBrito SR, Lee JJ, Caudle AS, Clemens MW, Gottumukkala VN, Katz MHG, Offodile AC, Uppal A; D3CODE Team, Chang GJ. Surgical Outcomes in Cancer Patients Undergoing Elective Surgery After Recovering from Mild-to-Moderate SARS-CoV-2 Infection. Ann Surg Oncol. 2021 Dec;28(13):8046-8053.
79. Kuznetsov D.V., Gevorgyan A.A., Novokshenov V.V., Kryukov A.V., Polyaeva M.V., Lyas M.N., Khalmetova A.A., Duplyakov D.V. Coronary artery bypass grafting in patients with coronary artery disease and COVID-19: search for an optimal strategy. *Russian Journal of Cardiology*. 2021;26(1S):4342. (In Russ.)
80. Mahrokhian SH, Nordan T, Ortoleva JP, Cobey FC, Chen FY, Kapur NK, Kawabori M. Successful use of Impella 5.5 to manage cardiogenic shock complicated by COVID-19. J Card Surg. 2021 Dec;36(12):4783-4785.
81. McPherson I, Chilvers N, Freystaetter K, Sivaharan A, Kanani M, Williams R, McCaslin J, Nandhra S, Booth K. Dissecting the Management and Outcomes of Thoracic Aortovascular Disease During the COVID-19 Pandemic. Ann Vasc Surg. 2021 Aug;75:120-127.
82. Mejia OAV, Borgomoni GB, Silveira LMV, Guerreiro GP, Falcão Filho ATG, Goncharov M, Dallan LRP, Oliveira MAP, de Sousa AG, Nakazone MA, Tiveron MG, Campagnucci VP, de Barros E Silva PGM, Dallan LAO, Lisboa LAF, Jatene FB; REPLICCAR Study Group. The arrival of COVID-19 in Brazil and the impact on coronary artery bypass surgery. J Card Surg. 2021 Sep;36(9):3070-3077.
83. Mohamed MO, Banerjee A, Clarke S, de Belder M, Patwala A, Goodwin AT, Kwok CS, Rashid M, Gale CP, Curzen N, Mamas MA. Impact of COVID-19 on cardiac procedure activity in England and associated 30-day mortality. Eur Heart J Qual Care Clin Outcomes. 2021 May 3;7(3):247-256.
84. Montandrau O, Arana H, Ehooman F, Bouattour K, Cherruault M, Ouechani W, Hamdaoui I, Dufour G, Kattou F, Philip I, Beaussier M. Surgical Revascularization With Cardiopulmonary Bypass on a Patient With Severe COVID-19. Semin Cardiothorac Vasc Anesth. 2021 Mar;25(1):46-50.
85. Nader J, Anselmi A, Tomasi J, Martin A, Aymami M, Rouze S, Corbineau H, Langanay T, Flecher E, Nesseler N, Verhoye JP. Adult cardiac surgery during COVID-19 lockdown: Impact on activity and outcomes in a high-volume centre. Arch Cardiovasc Dis. 2021 May;114(5):364-370.
86. Nguyen TC, Thourani VH, Nissen AP, Habib RH, Dearani JA, Ropski A, Crestanello JA, Shahian DM, Jacobs JP, Badhwar V. The Effect of COVID-19 on Adult Cardiac Surgery in the United States in 717 103 Patients. Ann Thorac Surg. 2022 Mar;113(3):738-746.
87. Özgür MM, Hançer H, Altaş Ö, Kırali K. COVID-19 ARDS in two patients with left ventricular assist device. J Artif Organs. 2021 Aug 1:1–7.
88. Parcha V, Kalra R, Glenn AM, Davies JE, Kuranz S, Arora G, Arora P. Coronary artery bypass graft surgery outcomes in the United States: Impact of the coronavirus disease 2019 (COVID-19) pandemic. JTCVS Open. 2021 Jun;6:132-143.
89. Ridwan K, DeVarennes B, Tchervenkov C, Shum-Tim D, Cecere R, Lachapelle K. Postoperative Nosocomial COVID-19 Infection in Cardiac Surgery: An Uncommon Event With High Mortality Rate. CJC Open. 2021 Oct;3(10):1217-1220.
90. Rodriguez-Leor O, Cid Alvarez AB, Pérez de Prado A, Rossello X, Ojeda S, Serrador A, López-Palop R, Martin-Moreiras J, Rumoroso JR, Cequier A, Ibáñez B, Cruz-González I, Romaguera R, Moreno R. In-hospital outcomes of COVID-19 ST-elevation myocardial infarction patients. EuroIntervention. 2021 Apr 20;16(17):1426-1433.
91. Rosati F, Muneretto C, Baudo M, D'Ancona G, Bichi S, Merlo M, Cuko B, Gerometta P, Grazioli V, Giroletti L, Di Bacco L, Repossini A, Benussi S. A multicentre roadmap to restart elective cardiac surgery after COVID-19 peak in an Italian epicenter. J Card Surg. 2021 Sep;36(9):3308-3316.
92. Perdoncin E, Greenbaum AB, Grubb KJ, Babaliaros VC, Keegan P, Ceretto-Clark B, Wei J, Guyton RA, Paone G, Byku I, Gleason PT, Biven K, Mathew P, Mortorano C, Inci EK, Faaborg-Andersen C, Mitchell R, Devireddy CM. Safety of same-day discharge after uncomplicated, minimalist transcatheter aortic valve replacement in the COVID-19 era. Catheter Cardiovasc Interv. 2021 Apr 1;97(5):940-947.
93. Smelt J, Santhirakumaran G, Vaughan P, Hunt I, Tan C. Thoracic Surgery during Coronavirus Disease 2019 (COVID-19): The Experience of a Level 1 Trauma Center. Thorac Cardiovasc Surg. 2021 Apr;69(3):252-258.
94. Tabaghi S, Akbarzadeh MA. Acute type A aortic dissection in a patient with COVID-19. Future Cardiol. 2021 Jul;17(4):625-629.
95. Taje, R., Elia, S., Cristino, B. *et al.* Thoracic surgery in the COVID-19 era: an Italian university hospital experience. *Cardiothorac Surg* **29,**21 (2021).
96. Uysal A, Erturk E, Abacilar AF, Duman U, Dogan OF. The Outcomes of Patients Incidentally Confirmed with Covid-19 After Cardiac Surgery. Heart Surg Forum. 2021 Nov 12;24(6):E940-E946.
97. Wang X, Gao H, Zhang Z, Deng C, Yan Y, Shi T. Effect of the COVID-19 pandemic on complications and mortality of patients with cardiac surgery. J Cardiothorac Surg. 2021 Dec 31;16(1):361.
98. Wójcik M, Gąsior A, Karpiak J, Chlebuś M, Romanek J, Rak M, Kolowca M, Widenka K, Przybylski A. Treatment outcomes of COVID-19 infected patients in bi-disciplinary cardiological and cardiac surgery ward. Kardiol Pol. 2021 Dec 31.
99. Zivkovic I, Milacic P, Mihajlovic V, Krasic S, Lesanovic J, Peric M, Zdravkovic D. Surgical treatment of ascending aorta floating thrombus in a patient with recent SARS-CoV-2 infection. Cardiovasc Diagn Ther. 2021 Apr;11(2):467-471.
100. Dib EP, Joseph S, Patel N, Rafael A, Meyer D, Bindra A, Hall S, Gong T. Left Ventricular Assist Device Implantation in a COVID-19 Positive Patient. J Heart Lung Transplant. 2021 Apr;40(4):S469–70.

**Wrong outcomes**

(outcomes not relevant, cannot be extracted)

1. Farrington, W. J. and Mack, C. A. and Karas, M. G. and Ivascu, N. S. and Robinson, N. B. and Iannacone, E. and Lau, C. and Mick, S. L. and Girardi, L. N. New York's perspective of COVID 19: Effect and impact on cardiac surgery
2. Perdoncin, E. and Greenbaum, A. B. and Grubb, K. J. and Babaliaros, V. C. and Keegan, P. and Ceretto-Clark, B. and Wei, J. E. and Guyton, R. A. and Paone, G. and Byku, I. and Glea. Safety of same-day discharge after uncomplicated, minimalist transcatheter aortic valve replacement in the COVID-19 era
3. Ahamed, J. Severe aortic stenosis patient risk during the COVID-19 pandemic
4. Arabi, A. and Ahmad, F. and Al-Suwaidi, J. and Al-Qahtani, A. and Asaad, N. and Rafie, I. and Patel, A. A. and El Hassan, M. A. M. The Impact of COVID-19 Outbreak on Cardiovascular Admissions
5. Casanova, J. and Pissarra, D. and Costa, R. and Salgueiro, E. and Pinho, P. Cardiothoracic surgery during the Covid-19 pandemic: Perioperative care, safety, and surgical results
6. Cereda, A. and Cugola, D. and Balestrieri, G. and Vassileva, A. and Valsecchi, O. Unexpected peak of mortality: The COVID-19 burden on Bergamo transcatheter aortic valve implantation register
7. Chitturi, K. R. and Faza, N. N. and Little, S. H. and Kleiman, N. S. and Reardon, M. J. and Goel, S. S. Transcatheter Mitral Valve Repair with MitraClip for Severe Mitral Regurgitation and Cardiogenic Shock During the COVID-19 Pandemic
8. Donatelli, F. and Miceli, A. and Glauber, M. and Cirri, S. and Maiello, C. and Coscioni, E. and Napoli, C. Adult cardiovascular surgery and the coronavirus disease 2019 (COVID-19) pandemic: the Italian experience
9. Donatelli, F. and Miceli, A. and Glauber, M. and Cirri, S. and Maiello, C. and Coscioni, E. and Napoli, C. The COVID-19 Pandemic and Acute Aortic Dissections in New York: A Matter of Public Health
10. Gregory, A. J. and Grant, M. C. and Boyle, E. and Arora, R. C. and Williams, J. B. and Salenger, R. and Chatterjee, S. and Lobdell, K. W. and Jahangiri, M. and Engelman, D. T. Cardiac Surgery-Enhanced Recovery Programs Modified for COVID-19: Key Steps to Preserve Resources, Manage Caseload Backlog, and Improve Patient Outcomes
11. Hassan, A. and Arora, R. C. and Adams, C. and Bouchard, D. and Cook, R. and Gunning, D. and Lamarche, Y. and Malas, T. and Moon, M. and Ouzounian, M. and Rao, V. and Rubens, F. and Tremblay, P Cardiac Surgery in Canada During the COVID-19 Pandemic: A Guidance Statement From the Canadian Society of Cardiac Surgeons
12. Hussain, Azhar and Balmforth, Damian and Yates, Martin and Lopez-Marco, Ana and Rathwell, Claire and Lambourne, Jonathan and Roberts, NThe Pan London Emergency Cardiac Surgery service: Coordinating a response to the COVID-19 pandemic
13. Khanna, S.The 3C’s: COVID-19, children, and cardiac surgery – do we know enough?
14. Khialani, B. and MacCarthy, P.Transcatheter management of severe aortic stenosis during the COVID-19 pandemic
15. In-Hospital Outcomes of Coronary Artery Bypass Grafting Candidates Undergoing Percutaneous Coronary Intervention during the Intensive Care Unit Restricted Covid-19 Pandemic: The Multi-Centre Prospective UK-REVASC Registry Kite, T. A. and Owens, C. and Chase, A. and Mozid, A. and Routledge, H. and Hoole, S. P. and Palmer, N. and Egred, M. and Perera, D. and Kyriacos, M. and
16. The impact of COVID-19 pandemic on congenital heart surgery practice: An alarming change in demographics Korun, O. and Yurdakök, O. and Arslan, A. and Çiçek, M. and Selçuk, A. and Kılıç, Y. and Altın, F. and Şaşmazel, A. and Aydemir, N. A.
17. Facilitating transcatheter aortic valve implantation in the era of COVID-19: Recommendations for programmes Lauck, S. and Forman, J. and Borregaard, B. and Sathananthan, J. and Achtem, L. and McCalmont, G. and Muir, D. and Hawkey, M. C. and Smith, A. and Høj
18. Impact of COVID-19 on cardiac procedure activity in England and associated 30-day mortality Mohamed, M. O. and Banerjee, A. and Clarke, S. and de Belder, M. and Patwala, A. and Goodwin, A. T. and Kwok, C. S. and Rashid, M. and Gale, C. P. and C
19. Commentary: Performing cardiac surgery in the coronavirus disease 2019 (COVID-19) era: What is the new normal? Mohammadi, S. and Kalavrouziotis, D.
20. Effects of COVID-19 pandemic on cardiac surgery practice in 61 Hospitals worldwide: results of a surveyOnorati, F. and Myers, P. and Bajona, P. and Perrotti, A. and Mestres, C. A. and Quintana, E.
21. COVID-19: The rising cost of cardiac surgery and disease Osman, F. and Caplin, N. and Bashir, M.
22. Prioritizing cardiovascular surgical care in COVID-19 pandemic: Shall we operate or defer? Patel, S. and Kaushik, A. and Sharma, A. K.
23. Cardiac Surgery During the Coronavirus Disease 2019 Pandemic: Perioperative Considerations and Triage Recommendations
24. Patel, V. and Jimenez, E. and Cornwell, L. and Tran, T. and Paniagua, D. and Denktas, A. E. and Chou, A. and Hankins, S. J. and Bozkurt, B. and Rosengart, T. K. and Jneid, H.
25. Cardiothoracic surgery during COVID-19: Our experience with different strategies Ralhan, S. and Arya, R. C. and Gupta, R. and W and er, G. S. and Gupta, R. K. and Gupta, V. K. and Bagga, S. and Mohan, B.
26. Characteristics and Outcomes of Patients Deferred for Transcatheter Aortic Valve Replacement Because of COVID-19 Ro, R. and Khera, S. and Tang, G. H. L. and Krishnamoorthy, P. and Sharma, S. K. and Kini, A. and Lerakis, S.
27. When to Consider Deferral of Surgery in Acute Type A Aortic Dissection: A Review Sabe, A. A. and Percy, E. and Kaneko, T. and Plichta, R. P. and Hughes, G. C.
28. Cardiac Surgery Conduct during COVID-19 Pandemic Salenger, R. and Etchill, E. W. and Ad, N. and Matthew, T. and Alejo, D. and Whitman, G. and Lawton, J. S. and Lau, C. L. and Gammie, C. F. and Gammie, J. S.
29. The Surge After the Surge: Cardiac Surgery Post–COVID-19 Salenger, R. and Etchill, E. W. and Ad, N. and Matthew, T. and Alejo, D. and Whitman, G. and Lawton, J. S. and Lau, C. L. and Gammie, C. F. and Gammie, J. S.
30. The impact of COVID-19 on the provision of cardiac surgical services Shafi, A. M. A. and Hewage, S. and Harky, A.
31. Elective cardiac surgery during the COVID-19 pandemic: Proceed or postpone? Shehata, I. M. and Elhassan, A. and Jung, J. W. and Urits, I. and Viswanath, O. and Kaye, A. D.
32. Managing Aortic Stenosis in the Age of COVID-19: Preparing for the Second Wave Sundt, T. M.
33. Cardiac surgery in the time of the novel coronavirus: Why we should think to a new normal Tamagnini, G. and Biondi, R. and Ricciardi, G. and Rutigliano, R. and Trias-Llimos, S. and Meuris, B. and Lamelas, J. and Del Giglio, M.
34. Managing Severe Aortic Stenosis in the COVID-19 Era Tanguturi, V. K. and Lindman, B. R. and Pibarot, P. and Passeri, J. J. and Kapadia, S. and Mack, M. J. and Inglessis, I. and Langer, N. B. and Sundt, T. M. and Hung, J. and Elmariah, S.
35. [Impact of COVID-19 pandemic on structural heart interventions in Italy] Tarantini, Giuseppe and Nai Fovino, Luca and Scotti, Andrea and Marchese, Alfredo and Berti, Sergio and Saia, Francesco and Gregori,
36. Ongoing transcatheter aortic valve implantation (TAVI) practice amidst a global COVID-19 crisis: nurse-led analgesia for transfemoral TAVI Vendrik, J. and de Boer, J. and Zwiers, W. and van Gilst, S. A. and Holierook, M. and Chekanova, E. V. and Henriques, J. S. and Baan, J.
37. Aortic stenosis in the time of COVID-19: Development and outcomes of a rapid turnaround TAVI service Aortic stenosis in the time of COVID-19: Development and outcomes of a rapid turnaround TAVI service
38. Minimally invasive cardiac valve surgery during the COVID-19 pandemic: to do or not to do, that is the question Fudulu, D. P. and Angelini, G. D. and Vohra, H. A.
39. Early outcomes of Stanford type A aortic dissection under the coronavirus disease 2019 (COVID-19) pandemic: A multicentre study from Hubei province Hu, X. and Wang, Y. and Liu, J. and Qiu, X. and Liu, X. and Jiang, X. and Huang, X. and Feng, X. and Zhang, Y. and Zhang, S. and Qian, H. and Liu, W. and Zhang, J. and Dong, J. and Chen, J. and Xia, J. and Dong, N. and Wu, L.
40. Adult cardiac surgery during COVID-19 lockdown: Impact on activity and outcomes in a high-volume centre Nader, J. and Anselmi, A. and Tomasi, J. and Martin, A. and Aymami, M. and Rouze, S. and Corbineau, H. and Langanay, T. and Flecher, E. and Nesseler, N. and Verhoye, J. P.
41. Transcatheter Aortic Valve Implantation During the COVID-19 Pandemic Valdebenito, M. and Massalha, E. and Barbash, I. M. and Maor, E. and Fefer, P. and Guetta, V. and Segev, A.
42. Successful Anesthesia Management in a Patient With Type A Aortic Dissection Complicated by Renal Failure and Suspected Coronavirus Disease Zheng, H. B. and Deng, Q. Z. and Chi, X. H. and Chen, M. B. and Xu, L. and Peng, Y. G. and Wan, L. and Fan, L. C.
43. COVID-19: An Argentinian perspective Benetti, F. and Del Prete, S. H. and Glanc, M. and Navia, D.
44. Why change? Lessons in leadership from the COVID-19 pandemic Ahlsson, A.
45. COVID-19 guidance for triage of operations for thoracic malignancies: A consensus statement from Thoracic Surgery Outcomes Research Network COVID-19 guidance for triage of operations for thoracic malignancies: A consensus statement from Thoracic Surgery Outcomes Research Network
46. Prognostic significance of cardiac injury in COVID-19 patients with and without coronary artery disease Barman, Hasan Ali and Atici, Adem and Sahin, Irfan and Alici, Gokhan and Aktas Tekin, Esra and Baycan, Omer Faruk and Ozturk, Fatih and
47. Management of elective aortic valve replacement over the long term in the era of COVID-19 Basman, C. and Kliger, C. A. and Pirelli, L. and Scheinerman, S. J.
48. Insertion technique for the Impella 5.0 heart pump in the COVID-19 era Berman, M. and Nachum, E. and Osman, M. and Hogan, J. and Hoole, S. and Catarino, P.
49. Low Incidence of SARS-CoV-2 in Patients with Solid Tumours on Active Treatment: An Observational Study at a Tertiary Cancer Centre in Bertuzzi, A. F. and Marrari, A. and Gennaro, N. and Cariboni, U. and Ciccarelli, M. and Giordano, L. and Quagliuolo, V. L. and Santoro, A.
50. Emergency hospital admissions and interventional treatments for heart failure and cardiac arrhythmias in Germany during the Covid-19 Bollmann, A. and Hohenstein, S. and Meier-Hellmann, A. and Kuhlen, R. and Hindricks, G.
51. Hospital admission rates, length of stay, and in-hospital mortality for common acute care conditions in COVID-19 vs. pre-COVID-19 era Butt, A. A. and Kartha, A. B. and Masoodi, N. A. and Azad, A. M. and Asaad, N. A. and Alhomsi, M. U. and Saleh, H. A. H. and Bertollini, R. and Abou-Samra, A. B.
52. Thoracic surgeons' insights: Improving thoracic surgery outcomes during the Coronavirus Disease 2019 pandemic Chen, K. N. and Gao, S. and Liu, L. and He, J. and Jiang, G. N. and He, J.
53. Committee Recommendations for Resuming Cardiac Surgery Activity in the SARS-CoV-2 Era: Guidance From an International Cardiac Surgery Consortium Chikwe, J. and Gaudino, M. and Hameed, I. and Robinson, N. B. and Bakaeen, F. G. and Menicanti, L. and Doenst, T. and Zheng, Z. and Lemma, M. and Falk, V. and Tatoulis, J. and Girardi, L. N. and Fremes, S. and Ruel, M.
54. Commentary: Coronavirus disease 2019 and acute aortic dissection: So many questions, so few answers Chu, M. W. A. and Ouzounian, M. and El-Hamamsy, I. and Peterson, M. D.
55. Restructuring Structural Heart Disease Practice During the COVID-19 Pandemic Chung, C. J. and Nazif, T. M. and Wolbinski, M. and Hakemi, E. and Lebehn, M. and Br and wein, R. and Rezende, C. P. and Doolittle, J. and Rabbani, L. and Uriel, N. and Schwartz, A. and Biviano, A. and Wan, E. and Hathaway, L. and Hahn, R. and Khalique, O. and Hamid, N. and Ng, V. and Patel, A. and Vahl, T. and Kirtane, A. and Bapat, V. and George, I. and Leon, M. B. and Kodali, S. K.
56. Emergency surgery in COVID-19 outbreak: Has anything changed? Single center experience D'Urbano, F. and Fabbri, N. and Koleva Radica, M. and Rossin, E. and Carcoforo, P.
57. Commentary: Cardiac surgery and coronavirus disease 2019 (COVID-19): Lessons learned in Italy-the hard way Duncan, A. E.
58. A Perspective from New York of COVID 19: Effect and impact on cardiac surgeryFarrington, W. J. and Mack, C. A. and Karas, M. G. and Ivascu, N. S. and Robinson, N. B. and Iannacone, E. and Lau, C. and Mick, S. L. and Girardi, L. N.
59. [Clinical feature changes of a COVID-19 patient from mild to critical condition and cardiopulmonary pathological results] Jiang, S. W. and Gao, H. and Wu, L. and Wang, G. W. and Cen, F. L. and Li, J. X. and Feng, C. and Wen, J. M. and Chen, Y. and He, R. L. and
60. Safety and operational efficiency of restructuring and redeploying a transcatheter aortic valve replacement service during the COVID-19 Joseph, J. and Kotronias, R. A. and Estrin-Serlui, T. and Cahill, T. J. and Kharb and a, R. K. and Newton, J. D. and Grebenik, C. and Dawkins, S. and Banning, A. P.
61. Fatal Post-Infarction Late Left Ventricular Free Wall Rupture in the Era of COVID-1 Kassimis, G. and Karagiannidis, E. and Triantafyllou, K. and Karapanagiotidis, G. T.
62. The impact of COVID-19 pandemic on cardiac surgery in Israel Keizman, E. and Ram, E. and Kachel, E. and Sternik, L. and Raanani, E.
63. Guidelines for Balancing Priorities in Structural Heart Disease During the COVID-19 Pandemic Khan, J. M. and Khalid, N. and Shlofmitz, E. and Forrestal, B. J. and Yerasi, C. and Case, B. C. and Chezar-Azerrad, C. and Musallam, A. and Rogers, T. and Waksman, R.
64. COVID-19-tailored approach to cardiac surgical safeguarding: stepwise protocol Khan, Y. and Mallick, H. and Shahabuddin, S.
65. [Consideration of surgeons participating in 2019 coronavirus disease emergency medical rescue] Li, S. and Li, X. D. and Wang, G. P. and Liang, C. and Jing, J. P. and Liu, M. M. and Zhang, C. and Lin, T. and Zhou, Y. H. and Song, Y. Z. and Tan, C. and Wang, Q. and Fan, L.
66. COVID-19 pandemic and admission rates for and management of acute coronary syndromes in England Mafham, M. M. and Spata, E. and Goldacre, R. and Gair, D. and Curnow, P. and Bray, M. and Hollings, S. and Roebuck, C. and Gale, C. P. and Mamas, M. A. and Deanfield, J. E. and de Belder, M. A. and Luescher, T. F. and Denwood, T. and L and ray, M. J. and Emberson, J. R. and Collins, R. and Morris, E. J. A. and Casadei, B. and Baigent, C.
67. Donation and transplantation activity in the UK during the COVID-19 lockdown Manara, A. R. and Mumford, L. and Callaghan, C. J. and Ravanan, R. and Gardiner, D.
68. Limitations of national database studies in cardiac surgery: Additional data required for individual risk stratification Martin, T. J. and Eltorai, A. E. M. and Kennedy, K. and Sellke, F. and Ehsan, A.
69. Assessment of Cardiovascular Events among Symptomatic Outpatients with Suspected Coronary Artery Disease Who Presented during COVID-19 Pandemic Mason, S. M. and Sekaran, N. and Ethington, J. D. and Nay, S. and Le, V. T. and Knowlton, K. U. and Meredith, K. G. and Min, D. B.
70. USING CORHEALTH CARDIAC REGISTRY DATA TO MONITOR AND COMPARE CARDIAC ACTIVITY ACROSS ONTARIO IN REAL TIME Oakes, G. and Jeffrey, J. and Pardhan, A. and Tang, J. and Jhaveri, R. and Leung, J. and Rahal, M. and Natarajan, M. and Woodward, G.
71. Minimising risk to thoracic surgical teams in an era of COVID-19: exploring possible preventative measures Patel, A. J. and Mohamed, S. and Caruana, E. J. and Naidu, B.
72. COVID-19 and ischemic heart disease emergencies: What cardiac surgery should expect? Pilato, E. and Manzo, R. and Comentale, G.
73. Solid organ transplantation programs facing lack of empiric evidence in the COVID-19 pandemic: A By-proxy Society Recommendation Consensus approachRitschl, P. V. and Nevermann, N. and Wiering, L. and Wu, H. H. and Moroder, P. and Br and l, A. and Hillebr and t, K. and Tacke, F. and Friedersdorff, F. and Schlomm, T. and Schöning, W. and Öllinger, R. and Schmelzle, M. and Pratschke, J.
74. Commentary: Echoes of war Rocco, G.
75. Cardiovascular care delivery during the second wave of COVID-19 in CanadaRoifman, I. and Arora, R. C. and Bewick, D. and Chow, C. M. and Clarke, B. and Cowan, S. and Ducharme, A. and Gin, K. and Graham, M. and Gupta, A. and Hardiman, S. and Hartleib, M. and Jackson, S. and Jassal, D. and Kazmi, M. and Lamarche, Y. and Légaré, J. F. and Leong-Poi, H. and Mansour, S. and Marelli, A. and Ruel, M. and Small, G. and Sterns, L. and Turgeon, R. and Virani, S. and Wijeysundera, H. C. and Wong, K. and Wood, D. A. and Zieroth, S. and Singh, G. and Krahn, A. D.
76. Mortality, Stroke, and Hospitalization Associated with Deferred vs Expedited Aortic Valve Replacement in Patients Referred for Symptomatic Severe Aortic Stenosis during the COVID-19 PandemicRyffel, C. and Lanz, J. and Corpataux, N. and Reusser, N. and Stortecky, S. and Windecker, S. and Pilgrim, T.
77. Delayed Diagnosis and Treatment of a Critically Ill Patient with Infective Endocarditis Due to a False-Positive Molecular Diagnostic Test for SARS-CoV-2Schizas, N. and Michailidis, T. and Samiotis, I. and Patris, V. and Papakonstantinou, K. and Argiriou, M. and Dedeilias, P.
78. Temporal trends in the presentation of cardiovascular and cerebrovascular emergencies during the COVID-19 pandemic in Germany: an analysis of health insurance claims Seiffert, M. and Brunner, F. J. and Remmel, M. and Thomalla, G. and Marschall, U. and L'Hoest, H. and Acar, L. and Debus, E. S. and
79. Cardiac manifestations in COVID-19 patients-A systematic review Shafi, A. M. A. and Shaikh, S. A. and Shirke, M. M. and Iddawela, S. and Harky, A.
80. Cardiovascular Interventions in Face of Covid-19 Pandemic in a Low to Middle Income Country of South Asia Region - A Pre and Post Covid Era Comparison! Shams, P. and Faheem, O. and Adnan, G. and Ali, J. and Khan, M.
81. Early Left Ventricular Thrombus Formation in a COVID-19 Patient with ST-Elevation Myocardial Infarction Shams, P. and Faheem, O. and Adnan, G. and Ali, J. and Khan, M.
82. Maintaining paediatric cardiac services during the COVID-19 pandemic in a developing country in sub-Saharan Africa: Guidelines for a scale up in the face of a global scale down Sokunbi, O. J. and Mgbajah, O. and Olugbemi, A. and Udom, B. O. and Idowu, A. and Sanusi, M. O.
83. Derivation and Validation of a Clinical Model to Predict Intensive Care Unit Length of Stay After Cardiac Surgery Sun, L. Y. and Bader Eddeen, A. and Ruel, M. and MacPhee, E. and Mesana, T. G.
84. COVID-19 Pandemics: A Surprising Link to Bread Flour With Collateral Damage to a Prosthetic Heart Valve Tozzi, P. and Kampouri, E. E. and Tzimas, G. and Prior, J. O. and Monney, P. and Kamani, C. and Lamoth, F.
85. The Netherlands Heart Journal: special issue on COVID-19 Vendrik, J. and Piek, J. J.
86. Safe Reintroduction of Cardiovascular Services During the COVID-19 Pandemic: From the North American Society LeadershipWood, D. A. and Mahmud, E. and Thourani, V. H. and Sathananthan, J. and Virani, A. and Poppas, A. and Harrington, R. A. and Dearani, J. A. and Swaminathan, M. and Russo, A. M. and Blankstein, R. and Dorbala, S. and Carr, J. and Virani, S. and Gin, K. and Packard, A. and Dilsizian, V. and Légaré, J. F. and Leipsic, J. and Webb, J. G. and Krahn, A. D.
87. Perioperative management of patients with suspected or confirmed COVID-19: review and recommendations for perioperative management from a retrospective cohort studyZheng, H. and Hébert, H. L. and Chatziperi, A. and Meng, W. and Smith, B. H. and Yan, J. and Zhou, Z. and Zhang, X. and Luo, A. and Wang, L. and Zhu, W. and Hu, J. and Colvin, L. A.
88. Clinical characteristics and prognosis of COVID-19 patients with initial presentation of lung lesions confined to a single pulmonary lobe Zhu, J. and Huang, W. C. and Huang, B. and Zhu, Y. and Jiang, X. J. and Zou, J. N. and Yang, G. and Wang, Z. and Ji, T. and Gu, M. M. and Zhou, X. and Gao, X. H.
89. Impact of SARS-CoV-2 outbreak on heart and lung transplant: A patient-perspective surveyBennett, D. and De Vita, E. and Ventura, V. and Bernazzali, S. and Fossi, A. and Paladini, P. and Luzzi, L. and Maccherini, M. and Valente, S. and Bargagli, E. and Frediani, B. and Sestini, P.
90. COVID-19-Associated Nonocclusive Fibrin Microthrombi in the Heart. Bois, M. C. and Boire, N. A. and Layman, A. J. and Aubry, M. C. and Alex and er, M. P. and Roden, A. C. and Hagen, C. E. and Quinton, R. A. and Larsen, C. and Erben, Y. and Majumdar, R. and Jenkins, S. M. and Kipp, B. R. and Lin, P. T. and Maleszewski, J. J.
91. Impact of the coronavirus disease 2019 (COVID-19) pandemic on the care of patients with acute and chronic aortic conditionsCzerny, M. and Gottardi, R. and Puiu, P. and
92. COVID-19 and the second wave during autumn: preventive strategies in cardiac and thoracic surgery divisions Donatelli, F. and Miceli, A. and Cirri, S. and Coscioni, E. and Napoli, C.
93. Lessons Learned from the Impact of the COVID-19 Pandemic in a Vascular Surgery Department and Preparation for Future Outbreaks Duarte, A. and Gouveia e Melo, R. and Lopes, A. and Rato, J. P. and Valente, J. and Pedro, L. M.
94. The triple-layer patch technique for post-infarction ventricular septal rupture Pacini, D. and Costantino, A. and Fiorentino, M. and Loforte, A. and Leone, A. and Botta, L.
95. Minimising risk to thoracic surgical teams in an era of COVID-19: exploring possible preventative measures Patel, A. J. and Mohamed, S. and Caruana, E. J. and Naidu, B.
96. How the COVID-19 pandemic changed treatment of severe aortic stenosis: a single cardiac center experience Perek, B. and Olasinska-Wisniewska, A. and Misterski, M. and Puslecki, M. and Grygier, M. and Buczkowski, P. and Lesiak, M. and Stankowski, T. and Szarpak, L. and Ruetzler, K. and Turan, O. and Jemielity, M.
97. Impact of Public Health Emergency Response to COVID-19 on Management and Outcome for STEMI Patients in Beijing-A Single-Center Historic Control Study Song, C. and Liu, S. and Yin, D. and Wang, Y. and Zhao, Y. and Yang, W. and Qiao, S. and Dou, K. and Xu, B.
98. The Feasibility and Safety of Routine Thoracic Surgeries in the Low-Risk Areas During the Coronavirus Disease 2019 Pandemic Wang, Y. and Zhang, Y. and Bao, F. and Hao, X. and Yu, F. and Lin, B. and Gu, Z. and Fang, W.
99. A retrospective approach to evaluating potential adverse outcomes associated with delay of procedures for cardiovascular and cancer-related diagnoses in the context of COVID-19 Zheng, N. S. and Warner, J. L. and Osterman, T. J. and Wells, Q. S. and Shu, X. O. and Deppen, S. A. and Karp, S. J. and Dwyer, S. and Feng, Q. and Cox, N. J. and Peterson, J. F. and Stein, C. M. and Roden, D. M. and Johnson, K. B. and Wei, W. Q.
100. Barkhordari, K. and Khajavi, M. R. and Bagheri, J. and Nikkhah, S. and Shirzad, M. and Barkhordari, S. and Kharazmian, K. and Nosrati, M. Early respiratory outcomes following cardiac surgery in patients with COVID-19
101. COVIDSurg Collaborative; GlobalSurg Collaborative. SARS-CoV-2 infection and venous thromboembolism after surgery: an international prospective cohort study. Anaesthesia. 2022 Jan;77(1):28-39.
102. Ad N, Luc JGY, Nguyen TC; COVID-19 North American Cardiac Surgery Survey Working Group. Cardiac surgery in North America and coronavirus disease 2019 (COVID-19): Regional variability in burden and impact. J Thorac Cardiovasc Surg. 2021 Sep;162(3):893-903.e4.
103. Aubert O, Yoo D, Zielinski D, Cozzi E, Cardillo M, Dürr M, Domínguez-Gil B, Coll E, Da Silva MI, Sallinen V, Lemström K, Midtvedt K, Ulloa C, Immer F, Weissenbacher A, Vallant N, Basic-Jukic N, Tanabe K, Papatheodoridis G, Menoudakou G, Torres M, Soratti C, Hansen Krogh D, Lefaucheur C, Ferreira G, Silva HT Jr, Hartell D, Forsythe J, Mumford L, Reese PP, Kerbaul F, Jacquelinet C, Vogelaar S, Papalois V, Loupy A. COVID-19 pandemic and worldwide organ transplantation: a population-based study. Lancet Public Health. 2021 Oct;6(10):e709-e719.
104. Benetti F, Del Prete SH, Glanc M, Navia D. COVID-19: An Argentinian perspective. J Card Surg. 2021 May;36(5):1717-1722
105. Nader J, Anselmi A, Tomasi J, Martin A, Aymami M, Rouze S, Corbineau H, Langanay T, Flecher E, Nesseler N, Verhoye JP. Adult cardiac surgery during COVID-19 lockdown: Impact on activity and outcomes in a high-volume centre. Arch Cardiovasc Dis. 2021 May;114(5):364-370.

**Wrong study type**

(non-observational)

1. Narayan, P. Cardiac surgery in presence of concomitant corona virus disease-2019 infection
2. Aghagoli, G. and Marin, B. G. and Soliman, L. B. and Sellke, F. W. Cardiac involvement in COVID-19 patients: Risk factors, predictors, and complications: A review
3. Ajibade, A. and Younas, H. and Pullan, M. and Harky, A. Telemedicine in cardiovascular surgery during COVID-19 pandemic: A systematic review and our experience
4. Bashir, M. and Moughal, S. Cardiovascular disease and surgery amid COVID-19 pandemic
5. Defilippis, E. M. and Sinnenberg, L. and Reza, N. and Givertz, M. M. and Kittleson, M. M. and Topkara, V. K. and Farr, M. A. Trends in US Heart Transplant Waitlist Activity and Volume during the Coronavirus Disease 2019 (COVID-19) Pandemic
6. Engelman, D. T. and Lother, S. and George, I. and Funk, D. J. and Ailawadi, G. and Atluri, P. and Grant, M. C. and Haft, J. W. and Hassan, A. and Legare, J. F. and Whitman, G. J. R. Adult Cardiac Surgery and the COVID-19 Pandemic: Aggressive Infection Mitigation Strategies Are Necessary in the Operating Room and Surgical Recovery
7. Fudulu, D. P. and Angelini, G. D. Cardiac surgery in the time of the coronavirus
8. Fudulu, D. P. and Angelini, G. D. and Vohra, H. The Pan London Emergency Cardiac Surgery service blueprint
9. Gaudino, M. and Chikwe, J. and Hameed, I. and Robinson, N. B. and Fremes, S. E. and Ruel, M. Response of Cardiac Surgery Units to COVID-19: An Internationally-Based Quantitative Survey
10. Hassan, A. and Arora, R. C. and Adams, C. and Bouchard, D. and Cook, R. and Gunning, D. and Lamarche, Y. and Malas, T. and Moon, M. and Ouzounian, M. and Rao, V. and Rubens, F. and Tremblay, P. and Whitlock, R. and Moss, E. and Légaré, J. F. Ramping Up the Delivery of Cardiac Surgery During the COVID-19 Pandemic: A Guidance Statement From the Canadian Society of Cardiac Surgeons
11. Hiremath, C. S. and Yadava, O. P. and Meharwal, Z. S. and Iyer, K. S. and Velayudhan, B. and Indian Assoc Cardiovasc Thoracic, S. IACTS guidelines: practice of cardiovascular and thoracic surgery in the COVID-19 era
12. Hiremath, Channabasavaraj Shivalingaiah and Yadava, Om Prakash and Meharwal, Zile Singh and Iyer, Krishna Subramony and Velayudhan, Bashi and Surg IACTS guidelines: practice of cardiovascular and thoracic surgery in the COVID-19 era
13. Jeyabalan, S. and Bangal, K. and Kulkarni, A. and Kavishree, M. and Kola, S. and Mahajan, M. and Okonna, F. and Nair, H. C. and S, K. M. and Kanchi, M.Anesthesia for Cardiac Surgery during COVID-19 Pandemic
14. Coronavirus Disease 2019 (COVID-19) and cardiac surgeon—Are we on the back foot? Khanna, S.
15. Safety for all: Coronavirus disease 2019 pandemic and cardiac surgery: A roadmap to 'phase' 2 Parolari, A. and di Mauro, M. and Bonalumi, G. and Barili, F. and Garatti, A. and Carretta, G. and Donato, D. and Pagano, D. and Gerosa, G.
16. Cardiac surgery during the COVID-19 pandemic: from vita minima to recovery Smail, H. and Stock, U. A. and De Robertis, F. and Bhudia, S. K. and Mittal, T. and Mattison, S. and Petrou, M. and Hill, J. and Gaer, J.
17. From Other Journals: A Review of Recent Articles in Pediatric Cardiology Aladağ, N. and Atabey, R. D.
18. TCT CONNECT-226 In-Hospital Outcomes of CABG Candidates Undergoing PCI During the ICU Restricted COVID-19 Pandemic: The Multi-Center Prospective UK-REVASC Registry Kite, T. and Ladwiniec, A. and Routledge, H. and Hoole, S. and Palmer, N. and Egred, M. and Perera, D. and Mouyis, K. and Sinha, M. and
19. Editor's Perspective: September 2020 Lau, J. W. Y.
20. The Evolving Pandemic of COVID-19 and Interventional Cardiology Mahmud, E.
21. Collateral damage of COVID-19 pandemic: Delayed medical care Masroor, S.
22. Solid-organ transplant surgeries in era of COVID-19 pandemic: How to go about it? Srivastava, D. and Solanki, S. and Ch and ra, A.
23. Cardiovascular Health in the COVID-19 Era: A Call for Action and Education Vallabhajosyula, S. and Friedman, P. A. and Bell, M. R.
24. Commentary: Coronary artery bypass grafting in patients with coronavirus disease 2019 (COVID-19): Darkness cannot drive out darkness Vervoort, D. and Nguyen, T. C.
25. Dilemma of organ donation in transplantation and the COVID-19 pandemicWoolley, A. E. and Mehra, M. R.
26. Congenital heart disease and incremental risks of COVID-19 Ogunjimi, M. and Haiduc, A. A. and Harky, A.
27. Best Practice Recommendations for Optimizing Care in Structural Heart Programs: Planning Efficient and Resource Leveraging Systems (PEARLS) Perpetua, E. M. and Guibone, K. A. and Keegan, P. A. and Palmer, R. and Speight, M. K. and Jagnic, K. and Michaels, J. and Nguyen, R. A. and Pickett, E. S. and Ramsey, D. and Schnell, S. J. and Wong, S. C. and Reisman, M.
28. COVID-19 in solid organ transplant recipients: A systematic review and meta-analysis of current literature Raja, M. A. and Mendoza, M. A. and Villavicencio, A. and Anjan, S. and Reynolds, J. M. and Kittipibul, V. and Fern and ez, A. and Guerra, G. and Camargo, J. F. and Simkins, J. and Morris, M. I. and Abbo, L. A. and Natori, Y.
29. Alizadeh Ghavidel, A. and Mirzaaghayan, M. and Yousefnia, M. A. and Asdaghpour, E. and Baghaei Tehrani, R. and Jalilifar, N. and Radmehr, H. and Shirzad, M. and Austine, N. Iranian Society of Cardiac Surgeons COVID-19 task force version II, restarting elective surgeries
30. Harky, A. and Chen, R. and Pullan, M. Examining the impact of COVID-19 on cardiac surgery services: The lessons learned from this pandemic
31. Harky, A. and Poole, G. and Axiaq, A. and Kirmani, B. H. COVID-19 and cardiac surgery: Do outcomes differ?
32. Hwang, D. and Zhan, Y. A Combination of Type A Aortic Dissection and COVID-19: Operative Mortality of 33%?
33. COVID-19 after heart transplant: Risk of severe progressions and mortality are probably significantly increased
34. Cardiothoracic robotic assisted surgery in times of COVID-19 Van den Eynde, J. and De Groote, S. and Van Lerberghe, R. and Van den Eynde, R. and Oosterlinck, W.
35. Six-Month Outcomes of Heart Transplant Recipients Infected by COVID-19 Diakos, N. and Latif, F. and Takeda, K. and Clerkin, K. and Habal, M. and Naka, Y. and Restaino, S. and Oh, K. and Sayer, G. and Farr, M. and Uriel, N.
36. Transcatheter aortic valve implantation versus surgical aortic valve replacement during the COVID-19 pandemic-Current practice and concerns Shafi, A. M. A. and Awad, W. I.
37. COVID-19: Yet another coronavirus challenge in transplantation Aslam, S. and Mehra, M. R.
38. Immediate impact of COVID-19 on transplant activity in the Netherlands de Vries, A. P. J. and Alwayn, I. P. J. and Hoek, R. A. S. and van den Berg, A. P. and Ultee, F. C. W. and Vogelaar, S. M. and Haase-Kromwijk, B. J. J. M. and Heemskerk, M. B. A. and Hemke, A. C. and Nijboer, W. N. and Schaefer, B. S. and Kuiper, M. A. and de Jonge, J.
39. Asymptomatic patients with coronavirus disease and cardiac surgery: When should you operate? Niknam, J. and Rong, L. Q.
40. Management Strategies for Patients After CABG Surgery in the Outbreak of the COVID-19 Pandemic Wang, X. G. and Zhou, Y. Z. and Zhang, X. and Li, J. M. and Zhou, X. M. and Zhang, Z. W.
41. Mechanical circulatory support-Challenges, strategies, and preparationsAwad, W. I. and Bashir, M.
42. COVID-19 in solid organ transplantation: an analysis of the impact on transplant activity and wait lists Bellini, M. I. and Tortorici, F. and Capogni, M.
43. Learn from the First Wave to Surf the Next One OptimallyChakfé, N. and Mertes, P. M. and Lejay, A.
44. Heart and Lung Transplantation in the Era of COVID-19: Early Recommendations and Outcomes Kapriniotis, K. and Giannis, D. and Geropoulos, G. and Evangeliou, A. P. and Ziogas, I. A. and Panagiotopoulos, N.
45. Rassaf, T. and Totzeck, M. and Mahabadi, A. A. and Hendgen-Cotta, U. and Korste, S. and Settelmeier, S. and Luedike, P. and Dittmer, U. and Herbstreit, F. and Brenner, T. and Klingel, K. and Hasenberg, M. and Walkenfort, B. and Gunzer, M. and Schlosser, T. and Weymann, A. and Kamler, M. and Schmack, B. and Ruhparwar, A. Ventricular assist device for a coronavirus disease 2019-affected heart.
46. Almeida RMS, Marin-Cuartas M, Garcia-Villarreal OA, Dayan V. COVID-19 and Cardiovascular Surgery. Do We Know What We Are Dealing With? Braz J Cardiovasc Surg. 2021 Dec 3;36(6):III-IV.
47. Chatterjee S, Coselli JS, Engelman DT. Commentary: "How to Slay the Aortic Dissection Beast in a COVID-19 World". Semin Thorac Cardiovasc Surg. 2021 Summer;33(2):313-315.
48. COVIDSurg Collaborative. Machine learning risk prediction of mortality for patients undergoing surgery with perioperative SARS-CoV-2: the COVIDSurg mortality score. Br J Surg. 2021 Nov 11;108(11):1274-1292.
49. Dhingra NK, Verma S, Yau TM, Yanagawa B, Hibino M. Stuck between a rock and a hard place: The clinical conundrum of managing cardiac surgical patients during the SARS-CoV-2 pandemic. J Card Surg. 2022 Jan;37(1):174-175.
50. Hirji SA, Zogg CK, Nguyen TC. Commentary: To operate or wait? Contextualizing early outcomes of cardiac surgery in COVID-19-positive patients. J Thorac Cardiovasc Surg. 2021 Aug;162(2):e373-e374.
51. Hwang D, Zhan Y. A Combination of Type A Aortic Dissection and COVID-19: Operative Mortality of 33%? Ann Thorac Surg. 2021 May;111(5):1734.
52. Lazzaro R, Patton B, Inra ML. Commentary: Coronavirus disease 2019 (COVID-19) and the thoracic surgeon: Choose wisely and preserve good judgment. J Thorac Cardiovasc Surg. 2021 Dec;162(6):1666-1667.
53. Obi K, Baldawi H, Garba S, Amoran O, Jenkins C, Gillies C, Penfold D, Dengle S, Alocozy L, Falloon A, Bob-Manuel T. Structural Heart Interventions During COVID-19. Curr Probl Cardiol. 2022 Feb;47(2):100934.
54. Okoh AK, Errine I, Soliman F, Johannesen J, D’Costa Z, Sethi A, Hakeem A, Chen C, Russo M. RAPID PERCUTANEOUS MITRAL VALVE REPAIR TREATMENT PROTOCOL DURING COVID-19 PANDEMIC. J Am Coll Cardiol. 2021 May 11;77(18):3155
55. Rosati CM, Nguyen CTN, Fukuhara S. COVID-19 and Aortic Dissections: Collaboration (Among Disciplines and Centers) Is Key. Ann Thorac Surg. 2021 Aug;112(2):683.
56. Rosati CM, Nguyen CTN, Fukuhara S. COVID-19 and Cardiac Surgery: Still Many Questions and Much Work to Do. Ann Thorac Surg. 2022 Feb;113(2):693-694.

Scarci M, Raveglia F. Commentary: Thoracic surgery in COVID-19 patients is not a taboo: A change of mind and correct timing are essential in COVID-19 surgical complications management. J Thorac Cardiovasc Surg. 2021 Dec;162(6):1665-1666.

**Full-text unavailable**

1. Barlow, C. W. Commentary: Cardiac surgery during the coronavirus disease 2019 (COVID-19) pandemic: Feeling our way in the dark
2. “Minimalist” transcatheter aortic valve implantation during the COVID-19 pandemic: Previously optional but now a necessity Wood, D. A. and Sathananthan, J.
3. Heart and infections Benamer, H. and Cayla, G.
4. ENCOURAGING OUTCOMES OF SOLID ORGAN RECIPIENTS WITH COVID-19 Bosch, F. and Borner, N. and Kemmner, S. and Lampert, C. and Jacob, S. and Koliogiannis, D. and Stangl, M. and Michel, S. and Kneidinger,
5. Commentary: Compliance with the American Association for Thoracic Surgery guidelines will prevent sternal wound infections and minimize postoperative complications in cardiac surgery patients during the COVID-19 pandemicLazar, H. L.
6. Retraction: Cardiovascular Disease, Drug Therapy, and Mortality in Covid-19. N Engl J Med. DOI: 10.1056/NEJMoa2007621 Mehra, M. R. and Desai, S. S. and Kuy, S. and Henry, T. D. and Patel, A. N.
7. T Bueser, T Clayton, M Dodd, E Beaumont, G Owens, S Murray, A Sepehripour, A Oo, J Sanders, The impact of COVID-19 on recovery after heart surgery: preliminary findings from the CardiacCovid Study, *European Journal of Cardiovascular Nursing*, Volume 20, Issue Supplement_1, July 2021, zvab060.051
8. Giovanni A. Chiariello, Piergiorgio Bruno, Natalia Pavone, Serena D’avino, Francesco Ferraro, Marialisa Nesta, Annalisa Pasquini, Federico Cammertoni, Piero Farina, Andrea Mazza, Federica Balducci, Maria Calabrese, Rocco A. Montone, Luca Montini, Massimo Massetti, 818 Impaired clinical outcome and increased postoperative complications in COVID-19 patients undergoing cardiopulmonary bypass, *European Heart Journal Supplements*, Volume 23, Issue Supplement_G, December 2021, suab149.010,
9. [Kaiche, R. K.](https://pesquisa.bvsalud.org/global-literature-on-novel-coronavirus-2019-ncov/?lang=pt&q=au:%22Kaiche,%20R.%20K.%22) Challenges of managing patients for urgent CABG in Covid times-Our Experience. *Anesthesia and Analgesia ; 133(3 SUPPL 2):318-319, 2021.*
10. Luckwell R, Luckwell R. P128 The implications of SARS-CoV-2 on a Cardiothoracic unit. BJS Open. 2021 Apr 8;5(Suppl 1):zrab032.127.

**Unable to extract data necessary for meta-analysis**

1. Solid Organ Transplantation in the Coronavirus Disease 2019 Era: “The Great Bet” in the North Italy Transplant Program Area Passamonti, S. M. and Cannavò, A. and Trunzo, V. and Caporale, V. and Buonocore, R. and DeFeo, T. M.
2. Cardiac Surgery Outcomes in an Epicenter of the COVID-19 Pandemic Farrington, W. J. and Robinson, N. B. and Rahouma, M. and Lau, C. and Hameed, I. and Iannacone, E. M. and Ivascu, N. S. and Mick, S. L. and Gaudino, M. F. and Girardi, L. N.
3. Barkhordari, Khosro, Mohamad R. Khajavi, Jamshid Bagheri, Sepideh Nikkhah, Mahmood Shirzad, Sepehr Barkhordari, Katayun Kharazmian, and Marjan Nosrati. "Early respiratory outcomes following cardiac surgery in patients with COVID‐19." *Journal of Cardiac Surgery* 35, no. 10 (2020): 2479-2485.
4. Fattouch, Khalil, Salvatore Corrao, Ettore Augugliaro, Alberto Minacapelli, Angela Nogara, Giulia Zambelli, Christiano Argano, and Marco Moscarelli. "Cardiac surgery outcomes in patients with coronavirus disease 2019 (COVID-19): A case-series report." *The Journal of Thoracic and Cardiovascular Surgery* (2020).
5. Garatti, Andrea, Serenella Castelvecchio, Andrea Daprati, Raffaella Molfetta, Marianna Volpe, Carlo De Vincentiis, Alessandro Parolari, and Lorenzo Menicanti. "Clinical Course of COVID-19 Infection in Patients Urgently Operated of Cardiac Surgical Procedures." *Annals of Surgery* 272, no. 4 (2020): e275-e279.
6. Knisely, Anne, Zhen Ni Zhou, Jenny Wu, Yongmei Huang, Kevin Holcomb, Alexander Melamed, Arnold P. Advincula et al. "Perioperative morbidity and mortality of patients with COVID-19 who undergo urgent and emergent surgical procedures." *Annals of Surgery* 273, no. 1 (2021): 34.
7. Lopez‐Marco, Ana, Amer Harky, Danilo Verdichizzo, Emma Hope, Barbara Rosser, Iain McPherson, Ronan Kelly, Luke Holland, Aung Ye Oo, and UK AS Research Group. "Early experience of aortic surgery during the COVID‐19 pandemic in the UK: A multicentre study." *Journal of Cardiac Surgery* 36, no. 3 (2021): 848-856.
8. Katsiampoura, Anastasia, Cesar Perozo, Andreas Varkaris, Sandhya Vellayappan, Ming Zhen Tam, Usha Vellayappan, Arvind Agnihotri, and Stanley Tam. "Covid‐19 positivity affects outcome of cardiac surgical patients." *Journal of Cardiac Surgery* 35, no. 12 (2020): 3650-3652
9. Mori, Makoto, Arnar Geirsson, Prashanth Vallabhajosyula, and Roland Assi. "Surgical management of thoracic aortic emergency with pre‐and postoperative COVID‐19 disease." *Journal of Cardiac Surgery* 35, no. 10 (2020): 2832-2834
10. Rassaf, Tienush, Matthias Totzeck, Amir A. Mahabadi, Ulrike Hendgen‐Cotta, Sebastian Korste, Stephan Settelmeier, Peter Luedike et al. "Ventricular assist device for a coronavirus disease 2019‐affected heart." *ESC heart failure* 8, no. 1 (2021): 162-166.
11. Sanders, Julie, Enoch Akowuah, Jackie Cooper, Bilal H. Kirmani, Mazyar Kanani, Metesh Acharya, Reuben Jeganathan et al. "Cardiac surgery outcome during the COVID-19 pandemic: a retrospective review of the early experience in nine UK centres." *Journal of Cardiothoracic Surgery* 16, no. 1 (2021): 1-10.
12. Garatti, A. and Castelvecchio, S. and Daprati, A. and Molfetta, R. and Volpe, M. and De Vincentiis, C. and Parolari, A. and Menicanti, L. Clinical Course of COVID-19 Infection in Patients Urgently Operated of Cardiac Surgical Procedures
13. Knisely, A. and Zhou, Z. N. and Wu, J. and Huang, Y. M. and Holcomb, K. and Melamed, A. and Advincula, A. P. and Lalwani, A. and Khoury-Collado, F. and Tergas, A. I. and St Clair, C. M. and Hou, J. Y. and Hershman, D. L. and D'Alton, M. E. and Huang, Y. Y. C. and Wright, J. D. Perioperative Morbidity and Mortality of Patients With COVID-19 Who Undergo Urgent and Emergent Surgical Procedures
14. Mori, M. and Geirsson, A. and Vallabhajosyula, P. and Assi, R. Surgical management of thoracic aortic emergency with pre- and postoperative COVID-19 disease.
15. COVIDSurg Collaborative; GlobalSurg Collaborative. Timing of surgery following SARS-CoV-2 infection: an international prospective cohort study. Anaesthesia. 2021 Jun;76(6):748-758.
16. Cardiothoracic Interdisciplinary Research Network and COVIDSurg Collaborative. Early outcomes and complications following cardiac surgery in patients testing positive for coronavirus disease 2019: An international cohort study. J Thorac Cardiovasc Surg. 2021 Aug;162(2):e355-e372.
17. Abbott TEF, Fowler AJ, Dobbs TD, Gibson J, Shahid T, Dias P, Akbari A, Whitaker IS, Pearse RM. Mortality after surgery with SARS-CoV-2 infection in England: a population-wide epidemiological study. Br J Anaesth. 2021 Aug;127(2):205-214.
18. Bonalumi G, Pilozzi Casado A, Barbone A, Garatti A, Colli A, Giambuzzi I, Torracca L, Ravenni G, Folesani G, Murara G, Pantaleo A, Picichè M, Villa E, Ferraro F, Vendramin I, Livi U, Montalto A, Musumeci F, Tarzia V, Trumello C, De Bonis M, Margari V, Paparella D, Salsano A, Santini F, Nicolardi S, Patanè F, Mammana L, Cura Stura E, Rinaldi M, Massi F, Triggiani M, Grazioli V, Giroletti L, Rubino A, De Feo M, Audo A, Regesta T, Barili F, Gerosa G, Di Mauro M, Parolari A. Prognostic value of SARS-CoV-2 on patients undergoing cardiac surgery. J Card Surg. 2022 Jan;37(1):165-173.
19. Chiariello GA, Bruno P, Pavone N, Calabrese M, D'Avino S, Ferraro F, Nesta M, Farina P, Cammertoni F, Pasquini A, Montone RA, Montini L, Massetti M. Bleeding Complications in Patients With Perioperative COVID-19 Infection Undergoing Cardiac Surgery: A Single-Center Matched Case-Control Study. J Cardiothorac Vasc Anesth. 2021 Nov 13:S1053-0770(21)00976-9.
20. Gomes WJ, Rocco I, Pimentel WS, Pinheiro AHB, Souza PMS, Costa LAA, Teixeira MMP, Ohashi LP, Bublitz C, Begot I, Moreira RSL, Hossne NA Jr, Vargas GF, Branco JNR, Teles CA, Medeiros EAS, Sáfadi C, Rampinelli A, Moratelli L Neto, Rosado AR, Mesacasa FK, Capriata IE, Segalote RC, Palmieri DLDRV, Jardim ACM, Vianna DS, Coutinho JHSA, Jazbik JC, Coutinho HMDR, Kikuta G, Almeida ZSM, Feguri GR, Lima PRL, Franco AC, Borges DC, Cruz FRH, Croti UA, Borim BC, Marchi CH, Goraieb L, Postigo KBS, Jucá FG, Oliveira FRA, Souza RB, Zilli AC, Mas RGS, Bettiati LC Junior, Tranchesi R, Bertini A Jr, Franco LV, Fernandes P, Oliveira F, Moraes R Jr, Araújo TCVN, Braga OP, Pedrosa AC Sobrinho, Teixeira RTB, Camboim ILL, Gomes EN, Reis PH, Garcia LP, Scorsioni NHG, Lago R, Guizilini S. COVID-19 in the Perioperative Period of Cardiovascular Surgery: the Brazilian Experience. Braz J Cardiovasc Surg. 2021 Dec 3;36(6):725-735.
21. Lazaros G, Oikonomou E, Theofilis P, Theodoropoulou A, Triantafyllou K, Charitos C, Charalambous G, Papanikolaou A, Gastouniotis I, Siasos G, Vlachopoulos C, Tousoulis D. The impact of COVID-19 pandemic on adult cardiac surgery procedures. Hellenic J Cardiol. 2021 May-Jun;62(3):231-233.
22. Mohamed MO, Curzen N, de Belder M, Goodwin AT, Spratt JC, Balacumaraswami L, Deanfield J, Martin GP, Rashid M, Shoaib A, Gale CP, Kinnaird T, Mamas MA. Revascularisation strategies in patients with significant left main coronary disease during the COVID-19 pandemic. Catheter Cardiovasc Interv. 2021 Dec 1;98(7):1252-1261.
23. [Monfared, M. B.](https://pesquisa.bvsalud.org/global-literature-on-novel-coronavirus-2019-ncov/?lang=pt&q=au:%22Monfared,%20M.%20B.%22); [Ghaderi, H.](https://pesquisa.bvsalud.org/global-literature-on-novel-coronavirus-2019-ncov/?lang=pt&q=au:%22Ghaderi,%20H.%22); [Aval, Z. A.](https://pesquisa.bvsalud.org/global-literature-on-novel-coronavirus-2019-ncov/?lang=pt&q=au:%22Aval,%20Z.%20A.%22); [Mirjafari, S. A.](https://pesquisa.bvsalud.org/global-literature-on-novel-coronavirus-2019-ncov/?lang=pt&q=au:%22Mirjafari,%20S.%20A.%22) Acute progressive arterial thrombosis after coronary artery bypass graft in a COVID-19 patient. *Immunopathologia Persa ; 8(1), 2022.*
24. Mountantonakis SE, Makker P, Saleh M, Coleman KM, Husk G, Jauhar R, Singh V, Epstein LM, Kuvin J. Increased Inpatient Mortality for Cardiovascular Patients During the First Wave of the COVID-19 Epidemic in New York. J Am Heart Assoc. 2021 Aug 17;10(16):e020255.
25. COVIDSurg Collaborative. Outcomes and Their State-level Variation in Patients Undergoing Surgery With Perioperative SARS-CoV-2 Infection in the USA: A Prospective Multicenter Study. Ann Surg. 2022 Feb 1;275(2):247-251.
26. [Reshetnikov, M. N.](https://pesquisa.bvsalud.org/global-literature-on-novel-coronavirus-2019-ncov/?lang=pt&q=au:%22Reshetnikov,%20M.%20N.%22); [Plotkin, D. V.](https://pesquisa.bvsalud.org/global-literature-on-novel-coronavirus-2019-ncov/?lang=pt&q=au:%22Plotkin,%20D.%20V.%22); [Zuban, O. N.](https://pesquisa.bvsalud.org/global-literature-on-novel-coronavirus-2019-ncov/?lang=pt&q=au:%22Zuban,%20O.%20N.%22); [Bogorodskaya, E. M.](https://pesquisa.bvsalud.org/global-literature-on-novel-coronavirus-2019-ncov/?lang=pt&q=au:%22Bogorodskaya,%20E.%20M.%22) EMERGENCY SURGICAL CARE FOR PATIENTS WITH COVID-19 AND TUBERCULOSIS COINFECTION AT MULTISPECIALTY HOSPITAL. *Bulletin of Russian State Medical University ; - (3):29-34, 2021.*
27. Sembiring YE, Puruhito P, Soebroto H, Prasmono A, Hakim AR, Sediono Pribadi OR, Suta Winarno DJ, Limanto DH, Akbar E, Ledyastatin RA, Agustio Putra Hutabarat MCB. Performing cardiac surgery during COVID-19 pandemic in Surabaya, Indonesia: A single-center retrospective observational study. Asian Cardiovasc Thorac Ann. 2021 Dec 17:2184923211066158.
28. Stammers AH, Mongero LB, Tesdahl EA, Patel KP, Jacobs JP, Firstenberg MS, Petersen C, Barletti S, Gibbs A. The assessment of patients undergoing cardiac surgery for Covid-19: Complications occurring during cardiopulmonary bypass. Perfusion. 2021 May 27:2676591211018983.
29. Vlastos D, Chauhan I, Mensah K, Cannoletta M, Asonitis A, Elfadil A, Petrou M, De Souza A, Quarto C, Bhudia SK, Rosendahl U, Pepper J, Asimakopoulos G. The impact of COVID-19 pandemic on aortic valve surgical service: a single centre experience. BMC Cardiovasc Disord. 2021 Sep 14;21(1):434.

**Appendix 4 – Risk of bias assessment for included studies using the Downs and Black checklist**

| **Study** | **Reporting ( /11)** | | | **External validity ( /3)** | | | **Bias ( /7)** | | | **Confounding ( /6)** | | | **Power ( /5)** | | | **Total score ( /32)** | | |
| --- | --- | --- | --- | --- | --- | --- | --- | --- | --- | --- | --- | --- | --- | --- | --- | --- | --- | --- |
|  | **Reviewer #1** | **Reviewer #2** | **Average** | **Reviewer #1** | **Reviewer #2** | **Average** | **Reviewer #1** | **Reviewer #2** | **Average** | **Reviewer #1** | **Reviewer #2** | **Average** | **Reviewer #1** | **Reviewer #2** | **Average** | **Reviewer #1** | **Reviewer #2** | **Average** |
| Fukuhara (2020)^12^ | **6** | **5** | **5.5** | **3** | **3** | **3** | **5** | **5** | **5** | **3** | **4** | **3.5** | **1** | **4** | **2.5** | **17** | **21** | **19** |
| Fukuhara (2020)^13^ | **8** | **7** | **7.5** | **3** | **3** | **3** | **5** | **5** | **5** | **3** | **4** | **3.5** | **5** | **5** | **5** | **19** | **24** | **21.5** |
| Hussain (2020)^14^ | **6** | **6** | **6** | **3** | **3** | **3** | **5** | **5** | **5** | **3** | **4** | **3.5** | **1** | **5** | **3** | **17** | **23** | **20** |
| Rescigno (2020)^15^ | **6** | **6** | **6** | **3** | **3** | **3** | **5** | **5** | **5** | **3** | **4** | **3.5** | **1** | **5** | **3** | **17** | **23** | **20** |
| Salna (2020)^16^ | **7** | **6** | **6.5** | **3** | **3** | **3** | **5** | **5** | **5** | **4** | **4** | **4** | **1** | **5** | **3** | **19** | **23** | **21** |
| Silveira (2020)^17^ | **7** | **6** | **6.5** | **3** | **3** | **3** | **5** | **5** | **5** | **4** | **4** | **4** | **1** | **5** | **3** | **19** | **23** | **21** |
| Varela Barca (2020)^18^ | **5** | **5** | **5** | **3** | **3** | **3** | **5** | **5** | **5** | **4** | **4** | **4** | **1** | **4** | **2.5** | **17** | **21** | **19** |
| Yandrapalli (2020)^19^ | **7** | **6** | **6.5** | **3** | **3** | **3** | **5** | **5** | **5** | **4** | **4** | **4** | **1** | **5** | **3** | **19** | **23** | **21** |
| Farsky (2021)^20^ | **6** | **6** | **6** | **3** | **3** | **3** | **5** | **5** | **5** | **3** | **4** | **3.5** | **2** | **5** | **3.5** | **17** | **23** | **20** |
| Romiti (2020)^21^ | **6** | **6** | **6** | **3** | **3** | **3** | **5** | **5** | **5** | **3** | **4** | **3.5** | **1** | **5** | **3** | **17** | **23** | **20** |
| Farina (2020)^22^ | **6** | **5** | **5.5** | **3** | **3** | **3** | **5** | **5** | **5** | **3** | **4** | **3.5** | **1** | **5** | **3** | **17** | **22** | **19.5** |
| Montandrau (2020)^23^ | **7** | **6** | **6.5** | **3** | **3** | **3** | **5** | **5** | **5** | **3** | **4** | **3.5** | **1** | **5** | **3** | **18** | **23** | **20.5** |
| Schwerzmann (2021)^24^ | **7** | **7** | **7** | **3** | **3** | **3** | **5** | **5** | **5** | **3** | **4** | **3.5** | **1** | **5** | **3** | **18** | **24** | **21** |
| Soetisna (2021)^25^ | **6** | **7** | **6.5** | **3** | **3** | **3** | **5** | **5** | **5** | **3** | **4** | **3.5** | **1** | **5** | **3** | **17** | **24** | **20.5** |
| Darvishi (2021)^26^ | **5** | **5** | **5** | **3** | **3** | **3** | **5** | **5** | **5** | **3** | **3** | **3** | **1** | **4** | **2.5** | **16** | **20** | **18** |
| Lopez-Marco (2021)^28^ | **7** | **8** | **7.5** | **3** | **3** | **3** | **5** | **4** | **4.5** | **3** | **3** | **3** | **3** | **5** | **4** | **18** | **23** | **20.5** |
| Keaton-Nasser (2021)^29^ | **5** | **3** | **4** | **3** | **3** | **3** | **5** | **5** | **5** | **3** | **3** | **3** | **1** | **4** | **2.5** | **16** | **18** | **17** |
| Omar (2021)^30^ | **7** | **7** | **7** | **3** | **3** | **3** | **5** | **4** | **4.5** | **3** | **3** | **3** | **3** | **5** | **4** | **18** | **22** | **20** |
